# Comparative integrated omics: identification of key functionalities in microbial community-wide metabolic networks

**DOI:** 10.1038/npjbiofilms.2015.7

**Published:** 2015-06-17

**Authors:** Hugo Roume, Anna Heintz-Buschart, Emilie E L Muller, Patrick May, Venkata P Satagopam, Cédric C Laczny, Shaman Narayanasamy, Laura A Lebrun, Michael R Hoopmann, James M Schupp, John D Gillece, Nathan D Hicks, David M Engelthaler, Thomas Sauter, Paul S Keim, Robert L Moritz, Paul Wilmes

**Affiliations:** 1 Luxembourg Centre for Systems Biomedicine, University of Luxembourg, Esch-sur-Alzette, Luxembourg; 2 Institute for Systems Biology, Seattle, WA, USA; 3 The Translational Genomic Research Institute-North, Flagstaff, AZ, USA; 4 Life Science Research Unit, University of Luxembourg, Luxembourg, Luxembourg

## Abstract

**Background:**

Mixed microbial communities underpin important biotechnological processes such as biological wastewater treatment (BWWT). A detailed knowledge of community structure and function relationships is essential for ultimately driving these systems towards desired outcomes, e.g., the enrichment in organisms capable of accumulating valuable resources during BWWT.

**Methods:**

A comparative integrated omic analysis including metagenomics, metatranscriptomics and metaproteomics was carried out to elucidate functional differences between seasonally distinct oleaginous mixed microbial communities (OMMCs) sampled from an anoxic BWWT tank. A computational framework for the reconstruction of community-wide metabolic networks from multi-omic data was developed. These provide an overview of the functional capabilities by incorporating gene copy, transcript and protein abundances. To identify functional genes, which have a disproportionately important role in community function, we define a high relative gene expression and a high betweenness centrality relative to node degree as gene-centric and network topological features, respectively.

**Results:**

Genes exhibiting high expression relative to gene copy abundance include genes involved in glycerolipid metabolism, particularly triacylglycerol lipase, encoded by known lipid accumulating populations, e.g., *Candidatus* *Microthrix parvicella*. Genes with a high relative gene expression and topologically important positions in the network include genes involved in nitrogen metabolism and fatty acid biosynthesis, encoded by *Nitrosomonas* spp. and *Rhodococcus* spp. Such genes may be regarded as ‘keystone genes’ as they are likely to be encoded by keystone species.

**Conclusion:**

The linking of key functionalities to community members through integrated omics opens up exciting possibilities for devising prediction and control strategies for microbial communities in the future.

## Introduction

Our ability to study microbial communities in natural settings as well as in engineered systems, e.g., biological wastewater treatment (BWWT) plants, has dramatically improved in recent years owing to rapid advances in high-throughput DNA sequencing technologies and other ‘meta-omic’ analyses which are driving molecular microbial ecology into the era of Eco-Systems Biology.^1^ Although metagenomic data provide gene inventories, without any proof of their functionality, the analysis of community-wide transcripts facilitates an assessment of community-wide functions,^2^ and community proteomics provide representation of the actual phenotypic traits of individual community members.^3^ Metabolomics, through resolving the final and intermediate products of cellular metabolism, should theoretically be the most sensitive indicator of community-wide phenotypes and allow inference of key metabolic processes.^4^ However, current metabolomic methodologies are limited in the number of metabolites that can be measured as well as their limited identifiability.^5^

The reconstruction of metabolic networks based on genomic data presents a compelling alternative to metabolomics for resolving the metabolic capabilities of organisms.^6^ So far, the conventional approach used to progress from single to multi-species metabolic network reconstructions has involved treating the metabolic networks of individual species as an input–output system to build network-based^7^ or constraint-based^8^ models of metabolic interactions. However, these multi-species models, which are usually limited to only a few species, fail to explain how variations in gene or species composition affect the overall metabolic state of ecosystems.^9^ Given the complexity of microbial communities, as well as the inability to isolate and sequence representative single cultures of all organisms within a community, such bottom-up approaches may be limited by the inherent impossibility to extrapolate community-wide networks and behaviour from individual isolate omic data sets.^1^ Recently developed alternative approaches involve the determination of community-wide metabolic potential^10^ and the reconstruction of community-wide metabolic networks based directly on metagenomic data,^11^ thereby ignoring the contribution of individual species.^12^ Through this population-independent approach, Greenblum *et al.*^12^ identified enzyme-coding genes, either enriched or depleted, in stool samples of human individuals with obesity or inflammatory bowel disease, highlighting the potential of such approaches for the identification of key metabolic traits within microbial consortia. Ideally, top-down and bottom-up approaches should be combined to identify links between microbial community structure and function, thereby bridging the gap between population-level metabolic networks and the larger community-wide networks to ultimately build a systems-level model of interactions between species.^13^

**Figure 1:**
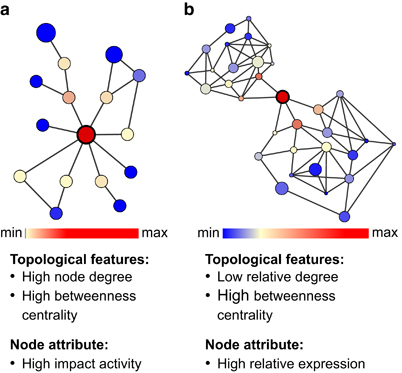
Criteria for defining keystone nodes in microbial species interaction and community-wide metabolic networks. (**a**) Criteria for identifying keystone species in reconstructed species interaction networks. Nodes represent taxa and edges represent associations between them. Node sizes reflect activity. (**b**) Criteria for identifying genes encoding key functionalities in reconstructed community-wide metabolic networks. Nodes represent enzyme-coding genes and edges correspond to shared metabolites (either reactants, products or educts). Node sizes reflect relative expression.

Here, we discuss a framework for comparative integrated omic analyses, which allows integration of systematically generated multi-omic data within reconstructed community-level metabolic networks. The resulting networks allow assessment of gene expression and protein abundances in combination with network topological features. We propose the use of these networks as an alternative to identifying keystone species through co-occurrence networks^14^ (Figure 1a). Reconstruction of co-occurrence networks requires large numbers of highly resolved samples and spurious correlations can affect interpretability of the resulting networks.^15^ Here, we identify genes encoding key functionalities in reconstructed community-wide metabolic networks and trace these back to the community members which encode them. Through their activity, keystone species are expected to have a disproportionately large effect on their environment, relative to their abundance.^16^ Their removal would greatly impact community structure and function.^17^ For example, in the human colon, specialist primary degraders such as *Ruminococcus bromii* are considered keystone species because of their ability to initiate the degradation of recalcitrant substrates.^18^ Herein, we define key functionalities as specific functions which have an overall pronounced effect on ecosystem functioning, because they exhibit a high relative gene expression and are represented by a node with a prominent topological position within a community-wide metabolic network (Figure 1b). The loss of such nodes would result in a lack of connectivity and this would greatly impact the overall topology of the community-wide metabolic network. In addition, the expression of these genes will likely be rate-limiting, similar to the effect of ‘load points’ on reconstructed single-organism metabolic networks,^19^ and thereby will govern the metabolic outcomes of the entire community. Therefore, by altering the expression of such genes, the community-wide phenotype could be influenced. By extension, members of the microbial community carrying out these functions would likely also be keystone species.

**Figure 2:**
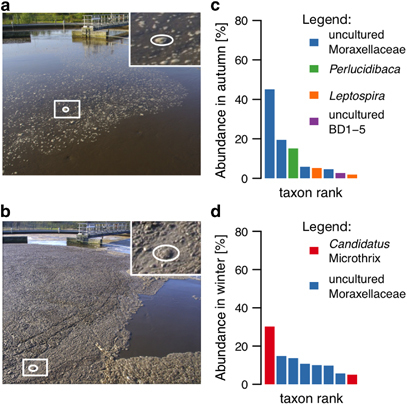
OMMC composition in autumn and winter seasons. Photographs of the OMMCs located at the water surface of the anoxic tank at the Schifflange BWWT plant in (**a**) autumn and (**b**) winter sampling dates. Abundance of genera of dominant community members based on reconstructed 16S rRNA gene sequences from metagenomic data in (**c**) autumn and (**d**) winter. OMMC, oleaginous mixed microbial community; rRNA, ribosomal RNA.

We apply the developed methodological framework to oleaginous mixed microbial communities (OMMCs) sampled from the surface of an anoxic BWWT tank in autumn and winter, respectively (Figure 2a,b). BWWT plants exhibit well-defined physical boundaries and represent a convenient and virtually unlimited source of spatially and temporally resolved samples. The microbial communities found in BWWT plants represent an ideal model system for microbial ecology^20^ because these communities are comparatively well described and lie between communities of low diversity, e.g., acid mine drainage biofilms,^21^ and complex communities such as those found in the human gastrointestinal tract^22^ or soil environments^23^ while retaining important hallmarks of both ends of the spectrum. These characteristics include (i) levels of dominance of individual taxa typically associated with low diversity communities (up to 30% of the community), most notably either *Candidatus* Microthrix parvicella (henceforth referred to as *Microthrix parvicella*) or *Perlucidibaca* spp. depending on the time of year;^24^ and (ii) the functional potential to adapt to rapid environmental changes typically observed in more diverse communities. Compared with BWWT microbial communities that are more typically studied, e.g., bulk activated sludge, OMMCs have additional important attributes which render them ideally suited as a model for the development and implementation of eco-systematic approaches. These include (i) limited species richness, i.e., operational taxonomic unit (OTU) richness of approximately 600 (Chao^25^ estimate from previous data^24^) compared with more than 1,000 (ref. 26) for activated sludge; (ii) high reproducibility between samples taken at the same time point.^4,27^ Apart from these characteristics, the targeted enrichment of OMMCs is of biotechnological interest as this would allow the reclamation of a significant fraction of the chemical energy contained within wastewater through lipid recovery and subsequent biodiesel synthesis.^28,29^ However, for such enrichment strategies to be successful, a detailed understanding of community function is necessary.^30^ For example, identified key functionalities may ultimately serve as driver nodes^31^ for controlling these communities.

## Materials and Methods

### Sampling

OMMCs were sampled from the anoxic tank of the Schifflange (Esch-sur-Alzette, Luxembourg; 49°30′48.29″N; 6°1′4.53″E) BWWT plant as described previously.^4^ Samples were taken on 4 October 2010 (referred to herein as the autumn OMMC) and 25 January 2011 (referred to herein as the winter OMMC; physico-chemical characteristics of the wastewater on the sampling dates are provided in Supplementary Table 1). These dates were chosen because they are representative of both extremes of OMMC-wide phenotypes, whereby, during the autumn sampling date, the tank exhibited only sparse amounts of OMMC biomass (Figure 2a) and, on the winter sampling date, ample amounts of OMMC biomass were present (Figure 2b).

### Biomolecular extractions

A previously developed biomolecular isolation framework for community-integrated omics^4,27^ was used to sequentially extract total RNA, genomic DNA and proteins from single OMMCs based on the Qiagen AllPrep DNA/RNA/Protein Mini kit (QA, Qiagen, Venlo, The Netherlands). The quality and quantity of isolated biomacromolecules were assessed as described previously^4^ (Supplementary Table 2, Supplementary Materials and methods).

### High-throughput sequencing

Total genomic DNA and ribosomal RNA-depleted retrotranscribed cDNA from both samples were sequenced on an Illumina Genome Analyzer IIx (Supplementary Materials and methods). Raw metagenomic and metatranscriptomic sequence data files are accessible in nucleic acid databases under BioProject PRJNA230567, sample LAO-A01 (SRX612782 and SRX612783) and LAO-A02 (SRX389533 and SRX389534).

### Metagenomic and metatranscriptomic sequence assembly, gene annotation and determination of gene abundances

Raw 100 nt paired-end sequencing reads from the metagenome and metatranscriptome libraries from each of the two sampling dates were first trimmed and quality filtered using the *trim-fastq.pl* script from the *PoPoolation* package^32^ and overlapping read pairs were assembled using the PAired-eND Assembler^33^ (*PANDAseq*). Non-redundant assembled *PANDAseq* read pairs and non-assembled reads from metagenomic and metatranscriptomic data sets of both sampling dates were then used as a single input for the *MOCAT* assembly pipeline.^34^ The resulting non-redundant contigs and *PANDAseq*-assembled read pairs that had not been used were then combined and filtered with a minimum length threshold of 150 bp. Protein-coding genes were predicted using the *Prodigal* gene finder^35^ (v2.60, contigs above 500 bp) or *FragGeneScan*^36^ (contigs between 150 and 500 bp). The resulting amino acid sequences from both contig sets were merged and made non-redundant using *CD-HIT.*^37^ All predicted gene sequences are accessible through *MG-RAST*^38^ as ID MGM4550606.3. The Kyoto Encyclopedia of Genes and Genome^39^ database version 64.0 was used to functionally annotate genes with Kyoto Encyclopedia of Genes and Genome orthologous groups (KOs) for ensuing metabolic network reconstruction (Supplementary Materials and methods, Supplementary Figure 1).

To allow meaningful comparisons between gene copy and transcript numbers from the two seasons, identical numbers of reads were sampled from the metagenomic and the metatranscriptomic libraries of both seasons (Supplementary Materials and methods) using an in-house developed Perl-script. The resulting reads were then mapped to the annotated gene sets. Cross-mapping reads were equally weighted according to the number of genes they mapped to and mapped reads were counted per gene. Finally, metagenomic and metatranscriptomic counts were normalised by the effective length of the gene sequence,^40^ yielding normalised gene copy abundances and normalised transcript abundances, respectively. KO abundances were inferred from the sums of normalised gene copy or transcript abundances of all genes belonging to a given KO (Supplementary Materials and methods). Relative gene expression values were determined per KO by calculating the ratio of normalised transcript abundances to normalised gene copy abundances (Supplementary Materials and methods, Supplementary Dataset 3).

### Metaproteome processing and analysis

Isolated and purified protein fractions were separated using one-dimensional SDS polyacrylamide gel electrophoresis. The proteins were reduced, alkylated, and digested with trypsin. The resulting peptides were then analysed by liquid chromatography coupled to tandem mass spectrometry. Peptide identification was carried out by database searching using the *X!Tandem* software^41^ with the amino acid sequence database generated from the genes predicted from the combined metagenomic and metatranscriptomic assembly. Protein identification was carried out using peptide-spectrum matches using the Trans-Proteomic Pipeline,^42^ with a probability of being correctly assigned to each protein determined by *PeptideProphet.*^43^ The protein inferences from each fraction were determined using *ProteinProphet* and then combined with *iProphet*^44^ to obtain a master set of identified proteins at a 1% false discovery rate. All proteomic data have been deposited in the PeptideAtlas mass spectrometry raw file repository at http://www.peptideatlas.org/PASS/PASS00512. Identified proteins were assigned KO numbers using BLAT-based^45^ alignment against the Kyoto Encyclopedia of Genes and Genome database v64.0 (Supplementary Materials and methods). Relative protein abundances were obtained using the normalised spectral index, as described previously^24^ (Supplementary Materials and methods, Supplementary Figure 4).

### Community-wide metabolic network reconstructions

Community-wide metabolic networks were reconstructed from the KOs with metabolic functions identified in the predicted gene sets from the combined metagenomic and metatranscriptomic assembly. The network reconstructions were rendered season-specific by using only KOs with mapped metatranscriptomic reads from each of the two sampling dates. The reconstructed networks reflect a connectivity-centred view of metabolism whereby enzymes grouped by KOs are represented by nodes and metabolites are represented by undirected edges, which represent either substrate or products of reactions catalysed by the respective KOs.^12^ Each KO was assigned a pair-set of substrate and product metabolites according to the RPAIR^46^ annotation in Kyoto Encyclopedia of Genes and Genome database version 67.1 (Supplementary Materials and methods).

### Topological network analysis and selection criteria for genes encoding key functionalities

To carry out a topological analysis of the reconstructed metabolic network, nodes and edges were rendered non-redundant, by representing multiple KOs with identical substrate and product metabolites as a single node. A comparison between the non-redundant network and a redundant version was also carried out (Supplementary Materials and methods). As most of the nodes that regroup several KOs represent subunits of the same enzyme, the small changes incurred on betweenness centrality and load by making the nodes non-redundant enhance the ability of these topological measures to identify key enzymes in the reconstructed community-wide metabolic networks (see also Supplementary Results and Discussion). Key functionalities were identified on the basis of topological criteria and relative gene expression. The topological selection criterion was defined in analogy to ‘load points’ as defined by Rahman and Schomburg^19^ in the context of reconstructed single-cell metabolic networks. Load points have the highest ratio of betweenness centrality (the number of valid shortest paths passing through them) relative to node degree (the number of neighbouring nodes; referred to as ‘neighbourhood connectivity’ by Rahman and Schomburg^19^). Node degree and betweenness centrality, among other topological measures, of each node were computed using the *Cytoscape Network-Analyzer* plug-in,^47^ considering the reconstructed network as undirected. These parameters were used to calculate load scores as defined in Equation (1).

(1)loadscoren=∑s≠n≠t(σst(n)/σst)kn∑e

where *s* and *t* are nodes in the network different from *n*, *σ*_*st*_ is the number of shortest paths from *s* to *t*, and *σ*_*st*_ *(n)* is the number of shortest paths from *s* to *t* that *n* lies on, *k*_*n*_ denotes the node degree of *n*, and Σ*e* denotes the total number of edges in the network. Thus, load score describes the number of reaction paths or conversions between metabolites that utilise a given enzyme, relative to its connectivity. It therefore serves as a proxy for an enzyme’s contribution to the metabolic fluxes of the overall community.

We prioritised the nodes with the top 10 per cent of load scores. In addition to this topological criterion, the relative gene expression of a node (either from a single KO or nodes regrouping several KOs) was also taken into account, such that only KOs with a high relative expression (top 10 per cent) were regarded as genes encoding key functionalities (Supplementary Materials and methods). Key functionalities were analysed for their involvement in the metabolism of uniquely occurring metabolites, i.e., to assess whether they represent ‘choke points’ as defined by Rahman and Schomburg.^19^ For the calculation of an alternative load score weighted according to the occurrence of the metabolites which should restrict ‘load points’ to nodes within pathways^46^ and a detailed analysis of sensitivity to the chosen cut-offs, see Supplementary Materials and methods.

### Linking genes encoding key functionalities to specific organisms

The presence of the identified genes in genomes of bacterial isolates was determined by aligning contigs bearing these genes to the contigs from genome assemblies of these strains using BLAST (Supplementary Materials and methods).

### Isolate strain culture and whole-genome sequencing

OMMC biomass sampled on 12 October 2011 was cultured on different growth media recommended for the culture of bacteria from water and wastewater and isolation procedures followed (Supplementary Materials and methods). In all, 140 pure bacterial cultures were obtained and screened for lipid inclusions using the Nile Red fluorescent dye.^48^ Following DNA extraction using the Power Soil DNA isolation kit (MO BIO, Carlsbad, CA, USA), the genomes of 85 Nile Red-positive isolates were sequenced on an Illumina HiSeq Genome Analyzer IIx using the same sequencing approach as described for the metagenomic samples. The resulting sequencing reads were assembled using either the *Abyss*^49^ or the *SPAdes*^50^ assemblers (Supplementary Materials and methods). Based on the presence of a gene encoding a key functionality, one isolate (Isolate LCSB065) was selected for refinement of genome assembly as well as phylogenetic and genomic analysis (Supplementary Materials and methods).

### Code availability and computational resources

All in-house developed scripts are available from the authors upon request. *In silico* analysis results were obtained using the high performance computing facilities of the University of Luxembourg.^51^

## Results and Discussion

### Identification of functions encoded and expressed in OMMCs in autumn and winter

High-resolution coupled metagenomic, metatranscriptomic and metaproteomic data were generated from the OMMCs sampled in autumn and winter. A total of 16.2 gigabases (Gb) of shotgun metagenomic paired-end 100 nt read sequence data as well as 38.6 Gb of metatranscriptomic sequence data were obtained. 6.5 million genes were predicted from a 6.7 million contigs of a combined assembly (1.6 Gb total length) of all metagenomic and metatranscriptomic reads (Supplementary Table 3). Based on reconstructed 16S ribosomal RNA gene sequences from the metagenomic data (Supplementary Materials and methods), the autumn and winter communities are dominated by *Perlucidibaca* spp. and *Microthrix* spp., respectively (Figure 2c,d, Supplementary Dataset 1). A total 830,679 predicted genes were annotated with KOs and regrouped (Materials and methods), yielding a total of 7,270 unique KOs. In the autumn sample, 10,074 protein groups (identified proteins grouped together because they share detected peptides) were identified using 19,248 non-redundant peptides out of a total of 727,155 mass spectra. In the winter sample, 7,106 protein groups were identified from 15,966 non-redundant peptides out of a total of 620,488 tandem mass spectra. A total 4,906 and 5,007 proteins were unambiguously identified in the autumn and winter samples, respectively.

**Figure 3:**
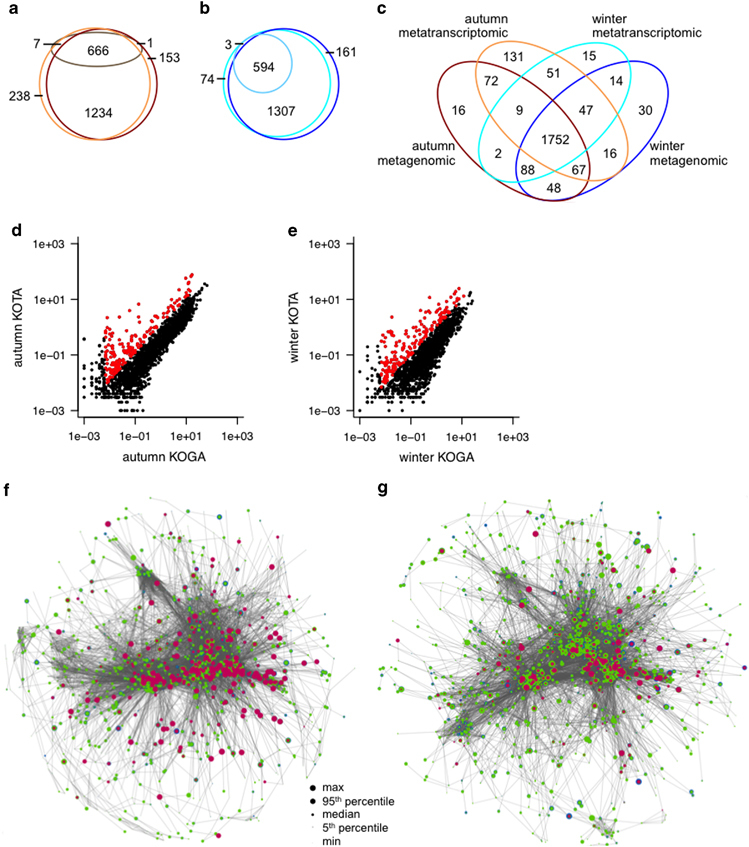
Integration of metagenomic, metatranscriptomic and metaproteomic data. (**a**) Venn diagram highlighting subsets of KEGG orthologous groups (KOs) in metabolic pathways present in the metagenomic (dark brown), metatranscriptomic (orange) and metaproteomic (pale brown) data from the autumn sample. (**b**) Subsets of KOs in metabolic pathways present in the metagenomic (dark blue), metatranscriptomic (cyan) and metaproteomic (pale blue) data from the winter sample. (**c**) Comparison of occurrence of KOs in metabolic pathways in metagenomic and metatranscriptomic data sets from autumn and winter. (**d**) Comparison of KO gene copy abundance (KOGA) and transcript abundance (KOTA) of KOs in metabolic pathways in the autumn data set. (**e**) Comparison of KO gene copy abundance (KOGA) and transcript abundance (KOTA) in metabolic pathways in the winter data set. In **d** and **e**, highly expressed KOs are highlighted in red. (**f**) Simplified autumn-specific metabolic network reconstruction. (**g**) Simplified winter-specific metabolic network reconstruction. In **f** and **g**, size of nodes represents KO abundance at metagenomic (blue), metatranscriptomic (green) and metaproteomic (magenta) levels, respectively. KEGG, Kyoto Encyclopedia of Genes and Genome.

The congruency between the metagenomic and metatranscriptomic data was high, as 92% of KOs represented in the metagenomic data are also present in the metatranscriptomic data for both autumn and winter data sets (Supplementary Dataset 2). The coverage of KOs was lower in the proteomic data, as 1,357 KOs (26% of KOs annotated in the metagenomic data set) and 1,236 KOs (23%) were identified in autumn and winter OMMCs, respectively. These proportions were mirrored by KOs within metabolic pathways (Figure 3a,b). This comparatively low metaproteomic coverage is due to current limitations in proteomic technologies for metaproteomic analyses.^52^

### Analysis of highly expressed genes in winter and autumn communities

Given the limited depth of coverage in the proteomic data, we mainly focused our subsequent comparative analyses on the metagenomic and metatranscriptomic data. Metaproteomic results were, however, used to corroborate and validate interpretations based on the analysis of the metatranscriptomic data. The comparison of KOs present in the metagenomic and metatranscriptomic data sets highlighted 757 (12%) and 210 (4%) unique KOs in autumn and winter OMMCs, respectively. Similar results were found in the comparison of KOs from metabolic pathways (Figure 3c). This analysis highlights a relatively limited difference in terms of genetic potential and gene expression between the two seasonally distinct OMMCs despite stark differences in community structure (Figure 2c,d).

For each identified KO, we calculated relative gene expression, which is considered to be more informative than simple transcript abundance because expression levels are normalised to metagenomic gene copy numbers.^53^ Furthermore, it allows quantitative insights into the contribution of low abundance members (such populations may be potential keystone species) to overall community activity to be obtained.^54^ KOs with high relative expression in both seasons (Figure 3d,e, Supplementary Dataset 3) were further analysed, as these are good candidates for genes which likely affect the overall community phenotype. Among these, enrichments were found in KOs linked to nitrogen metabolism, as well as oxidative phosphorylation and non-ribosomal peptide synthesis in both seasons (Supplementary Dataset 3). The highly expressed KOs involved in nitrogen metabolism represent enzymes for ammonium assimilation and oxidation, denitrification and nitrification. In particular, they include genes encoding likely subunits of ammonia monooxygenase (AMO; K10944, K10945 and K10946). AMO has a key role in the first step of nitrification carried out by aerobic ammonia-oxidising bacteria, mainly belonging to *Nitrosomonas* spp. and *Nitrosospira* spp.^54^ AMO was previously found to be highly expressed in BWWT biomass.^55^ In addition to the nitrogen metabolism enzymes expressed at a high level in both seasons, a nitrite reductase gene (K00363) was highly expressed in the autumn sample.

In the winter sample, the glycerolipid metabolism was enriched within highly expressed KOs. In particular, triacylglycerol lipase (K01046) exhibited pronounced transcript levels and its expression was also confirmed at the protein level (Supplementary Dataset 2). The most highly expressed genes of the 6,222 genes belonging to this KO could be matched to *Acinetobacter* spp., which are known to occur in BWWT plants and accumulate triacylglycerols.^56^ Furthermore, out of the genes with detectable expression, the two gene sequences with the highest gene copy numbers (i.e., abundance in the metagenomic data) were matched to the genome sequence of *Microthrix parvicella* BIO17-1 (ref. 57), which is enriched in KOs involved in lipid metabolism^57^ (11.3% of its annotated genes). The presence of these enzymes was recently suggested to be essential for lipid accumulation in a metabolic model reconstruction of *Microthrix parvicella,*^58^ but not until now were they found to be expressed in biological wastewater treatment communities. The pronounced expression of the aforementioned KOs involved in ammonium oxidation and the hydrolysis of triacylglycerols during both seasons emphasises the capability of the OMMCs to remove two of the main compounds present in wastewater, i.e., ammonia^59^ and lipids.^60^

In the winter sample, KOs from the TCA cycle were also strongly expressed and the majority could be detected at the proteome level. Rather surprisingly, in the autumn sample, photosynthesis KOs were enriched. Expression of photosystem I in autumn was also confirmed by proteomics suggesting that phototrophic organisms are part of the floating OMMC during this season.

### Reconstruction of a generalised and season-specific OMMC-wide metabolic networks

A community-wide metabolic network was reconstructed using the KOs expressed in the autumn and winter samples (Materials and methods, Supplementary Figure 5, Supplementary Dataset 4). The reconstructed network comprised 1,432 KO nodes with 29,988 edges representing non-unique metabolites.

Season-specific networks were reconstructed analogous to the generalised OMMC-wide network, but by only using the 1,885 KOs or 1,775 KOs expressed in autumn or winter, respectively (Figure 3f,g, Supplementary Datasets 5 and 6). This yielded networks comprising 1,298 nodes with 25,842 edges and 1,375 nodes with 27,370 edges forming a connected network for winter and autumn, respectively.

Among the KOs specific to the autumn network, functions in the metabolic pathways for porphyrin and chlorophyll metabolism, sesquiterpenoid, triterpenoid and carotenoid biosynthesis pathways (ko00860, ko00909 and ko00906) were found to be enriched. This reinforces the notion that photosynthesis occurs in the OMMC sampled in autumn, while photosynthetic gene appear to be below the detection limit in the winter sample.

### Identification of season-specific metabolic traits

The autumn- and winter-specific community-wide metabolic network reconstructions exhibit similar structures (Figure 3f,g) and represent 1,605 common KOs (i.e., 88 or 94% of the KOs included in the autumn or winter network reconstructions, respectively). Based on the reconstructed networks, a detailed network topological analysis was carried out (Supplementary Dataset 7).

Load scores (Equation 1) were determined in the reconstructed season-specific community-wide metabolic networks (Materials and methods). Most of the nodes in both the autumn- and winter-specific networks, which feature a high degree, represent KOs involved in amino acid synthesis. The relative small average shortest path lengths of 3.21 and 3.29 in the autumn and winter network reconstructions demonstrate that these represent ‘small world’ networks.^61^ Among the nodes with the highest betweenness centrality, i.e., the highest number of shortest paths passing through a node,^62^ in both metabolic reconstructions, KOs with functions in pyruvate metabolism, glycolysis or gluconeogenesis and glycerolipid metabolism were enriched (false discovery rate-adjusted *P* value <0.05). In contrast, relatively higher betweenness centrality of the nodes representing KOs in fatty acid metabolism pathway (ko01212) was observed in the network reconstruction from the winter data set (median fold change of 4; Wilcoxon signed rank test *P* value <0.001; enriched with false discovery rate-adjusted *P* value <0.00001; Supplementary Figure 6, Supplementary Dataset 7) suggesting distinct substrate usage in both seasons. Other functions, in which this subset of KOs was enriched, included porphyrin and chlorophyll metabolism, biotin metabolism, polyketide sugar unit biosynthesis, lipoic acid metabolism and fluorobenzoate degradation (ko00860, ko00780, ko00523, ko00785 and ko00364), while only phosphoinositol metabolism (ko00562) was significantly enriched among the functions of the nodes with a higher betweenness centrality in the autumn network.

### Identification of genes encoding key functionalities

**Figure 4:**
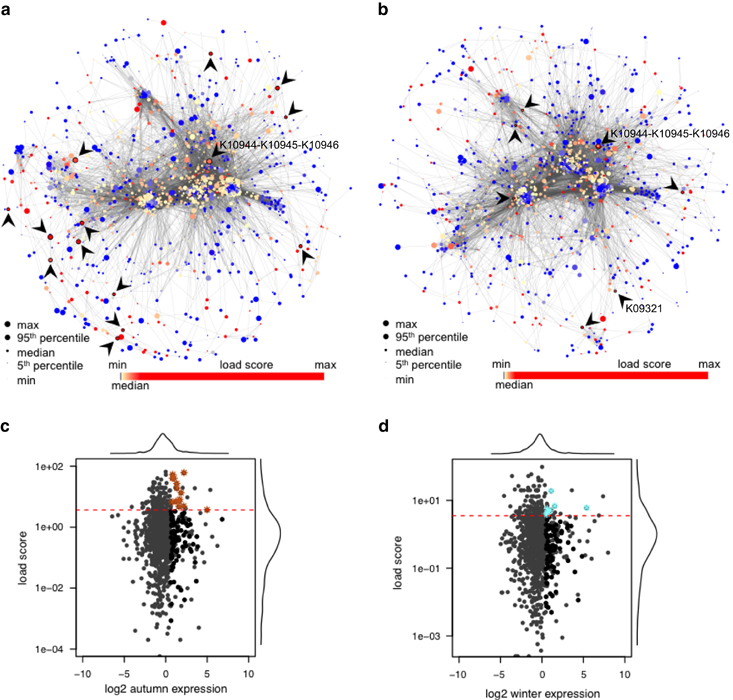
Topological analysis of the reconstructed season-specific community-wide metabolic networks and assessment of relative gene expression. (**a**) Autumn- and (**b**) winter-specific networks. In (**a**) and (**b**) node colours refer to *load score* and node sizes represent relative gene expression. KOs encoding key functionalities are encircled and highlighted by arrow heads. (**c** and **d**) Results of the topological analysis of KOs in simplified season-specific networks for (**c**) autumn and (**d**) winter. Highly expressed genes are indicated as black dots and KOs encoding key functionalities are indicated by brown (autumn) or cyan (winter) asterisks. Dotted red lines indicate minimal *load score* of KOs deemed to encode key functionalities.

Keystone species occupy topologically important positions in species interaction networks^63^ and are characterized by a high relative activity.^17^ Within a community-wide metabolic network reconstruction, key functionalities contributed by keystone populations should be encoded by genes which exhibit a high relative gene expression and these genes should also occupy important topological positions in relation to the community-wide metabolic network, i.e., they should represent ‘load points’^19^ (Figure 1b). Herein, we therefore identify genes having a high load score (Equation 1) within the season-specific metabolic networks as well as high relative expression in the respective data sets (Figure 2b, Figure 4, Materials and methods). Selected genes are reported and potential ‘choke points’ are indicated in Supplementary Dataset 7. According to Rahman and Schomburg, choke points are special cases of load points, which consume and/or produce unique metabolites. Given that uniqueness of a metabolite is a strong claim in the context of the reconstructed community-wide metabolic networks as much of community metabolism remains unknown (only 13% of the predicted genes could be confidently annotated with a function), the identification of key functionalities by using load points was chosen as a more robust and appropriate measure in the present case. The positions of the key functionalities within the networks as per our criteria (Figure 1b) are indicated in Figure 4 and Supplementary Figure 7. KOs involved in porphyrin and chlorophyll metabolic pathways are enriched among the selected genes in the autumn community, as are KOs with a function in degradation of aromatic compounds. Among the genes encoding key functionalities in the winter OMMCs, no significant enrichment among KOs from a particular pathway could be observed. However, one of these genes is K03921, coding for an acyl-[acyl-carrier-protein] desaturase, which is part of the biosynthesis pathway for polyunsaturated fatty acids.

In both the autumn and winter sets of season-specific key genes, the subunits of ammonia or methane monooxygenase (AMO or MMO) stand out. As discussed above and given the sampling from a nitrifying–denitrifying wastewater treatment plant, this is likely an AMO which catalyses the first essential step of nitrification by converting ammonia to hydroxylamine.^64^ In contrast, MMO is involved in methane oxidation, which is less likely to be expressed in the sampled environment.

### Linking genes encoding key functionalities to community members

Having selected genes encoding key functionalities within the sampled OMMCs using the reconstructed community-wide metabolic networks (Supplementary Dataset 7), we were interested in revealing which organisms expressed these genes within the community. As these genes contribute essential functionalities to the community and are characterized by relatively high expression, they are likely to be encoded by keystone species. Contigs containing genes annotated with one of the genes encoding key functionalities were selected from the combined metagenomic and metatranscriptomic data sets. These contigs were aligned to the NCBInr nucleotide database (Supplementary Dataset 7) to identify organisms encoding genes with similarity to the expressed genes of interest.

For five such genes (K03921, K01186, K01576, K01709 and K03335), no significant matches could be identified. On the other hand, three of these key genes from the winter-specific network (K01251, K00789 and K03527) were expressed from a multitude of contigs, which could be aligned well to over 50 different species. Half of the matched contigs encoding the five autumn key genes from the chlorophyll- and porphyrin-synthesis pathway (K03403, K03404, K03405, K04034, K04035) were most similar to sequences encoded by the genome of the cyanobacterium *Oscillatoria nigro-viridis* PCC 712. The relative expression of these genes accounted for 85% of the expression of these genes in autumn (Supplementary Dataset 7). Some *Oscillatoria* spp. are found in wastewater, where they have been found to participate in nitrate removal.^65^

**Figure 5:**
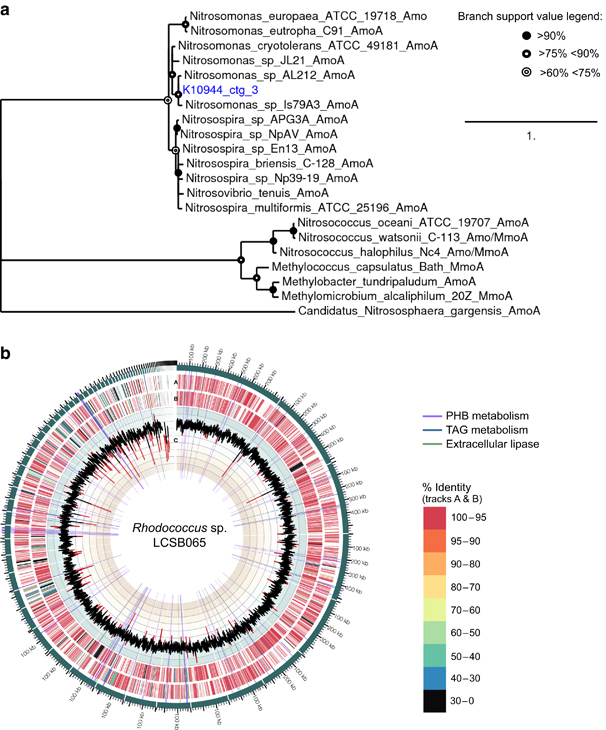
Linking key functionalities to important community members. (**a**) Phylogenetic tree based on the AmoA amino acid sequence derived from a contig extended using combined metagenomic and metatranscriptomic data (K10944_ctg_3). (**b**) Circos plot of the genome of Isolate LCSB065, highlighting amino acid similarity of encoded proteins to the *Rhodococcus erythropolis* PR4 genome and genes involved in poly-hydroxybutyrate (PHB) and TAG accumulation as well as encoded extracellular lipases. From the outside to the inside track: contigs (green) arranged by size; A: open reading frames in forward direction; B: open reading frames in reverse direction; colours in tracks A and B indicate %-similarity to the *Rhodococcus erythropolis* PR4 genome; C: %G+C in 1,000 bp sliding windows. Highlighted rays indicate the location of genes involved in PHB metabolism (violet), genes involved in TAG metabolism (blue) and extracellular lipase genes (green). TAG, triacylglycerol.

From the list of genes encoding key functionalities, we further selected the acyl-[acyl-carrier protein] desaturase (K03921) and the three subunits of AMO or MMO (K10944, K10945 and K10946) for further analysis. In all, 922 out of 1,067 contigs belonging to the AMO or MMO complex matched best to sequences of *Nitrosomonas* spp. a well-known genus of nitrifiers. The other contigs matched sequences from uncultured organisms or, in two cases, to a MMO from *Methylovulum miyakonense*. These two cases only represented 0.1% of the total contig length of the KOs K10944–K10946. Furthermore, less than 1% of the metatranscriptomic reads mapped to these two contigs, suggesting that the major function of these KOs is in ammonia rather than methane oxidation. In addition, a refined assembly of contigs belonging to K10944–K10946 using additional metagenomic data from a third sampling date (Supplementary Materials and methods) yielded a new contig containing complete sequences for *amoA* (an established phylogenetic marker for nitrifying microorganisms^66^), and *amoB*, both also matching best to *Nitrosomonas* spp. A phylogenetic tree was reconstructed using the predicted amino acid sequence of *AmoA* from this contig and the tree clearly places it closest to sequences of *Nitrosomonas* spp. (Figure 5a, Supplementary Table 4). To estimate the abundance of *Nitrosomonas* spp. in the sampled OMMCs, metagenomic and metatranscriptomic reads were mapped against the genome sequence of *Nitrosomonas* sp. Is79 (ref. 67), yielding approximately twice as many metagenomic reads in winter compared with autumn (Supplementary Table 5). The ratio of metatranscriptomic to metagenomic coverage was four times higher in winter than in autumn, indicating a higher general level of activity of *Nitrosomonas* spp. in the winter OMMC, although AMO activity was high in both seasons.

In contrast to the compelling link between the putative AMO genes and *Nitrosomonas* spp., linking the acyl-[acyl-carrier protein] desaturase unambiguously to an organismal group could not be achieved by simple alignment to reference genomes in public databases. Of the 14 contigs which harboured genes annotated with K03921 expressed in the winter sample, 9 did not yield any hits with a percentage identity >80% and query coverage >50%. The remaining five contigs yielded hits with 82 to 86% identity to sequences from *Rhodococcus erythropolis, Amycolatopsis mediterranei* and *Nocardia cyriacigeorgica*. As none of these alignments were of high confidence, we aligned the contigs encoding acyl-[acyl-carrier protein] desaturases to genomes of an in-house bacterial isolate collection from the same BWWT plant. Three of the contigs containing expressed genes matched to the same gene of the genome of Isolate LCSB065 with 88 to 100% identity over a total of 459 nt of the combined metagenomic contig length of 678 nt. Isolate LCSB065’s 81 contigs contain an almost complete 7.67 Mbp genome with a GC-content of 62.4% (Figure 5b, Supplementary Dataset 8). Based on the use of 31 bacterial protein coding marker genes, this isolate was identified as a *Rhodococcus* sp.^68^ (Supplementary Dataset 8). A detailed genomic analysis revealed a high number of genes involved in lipid metabolism encoded by this organism (Supplementary Results and Discussion) and non-polar storage granules were also observed microscopically (Supplementary Figure 8). As *Rhodococcus* spp. are known to exhibit lipid accumulation phenotypes,^69^ it is likely that this organism is a keystone species within the OMMC. Recruitment of metagenomic and metatranscriptomic reads to the isolate’s genome (Supplementary Dataset 8) revealed a low abundance of this organismal group in both autumn and winter, with a relative high transcriptional activity only in winter (Figure 5b, Supplementary Table 5) potentially directly linking its activity to the high community-wide lipid accumulation phenotype observed in winter.^24^ Low abundance combined with an activity with a great impact on their environment are hallmarks of keystone species and the *Rhodococcus* population fulfils these criteria in the context of the sampled OMMC.

## Conclusion

Despite stark differences in the appearance and structure of the sampled autumn and winter OMMCs, the comparative analysis of integrated metagenomic, metatranscriptomic and metaproteomic data contextualised in reconstructed community-wide metabolic networks uncovered surprisingly few global differences in terms of functional genetic potential and gene expression between the two communities. This result confirms previous observations that taxonomic profiles can be very variable whereas global functional profiles are typically more conserved.^70,71^ Nonetheless, our approach highlighted genes coding for essential enzymes involved in nitrogen metabolism (genes involved in nitrification, denitrification and ammonium oxidation) as being relatively highly expressed in both seasons despite exhibiting only low gene copy numbers. Identified differences between the two seasons include a marked expression of enzymes involved in glycerolipid metabolism in winter when OMMC biomass is most pronounced (Figure 2a,b) and lipid accumulation is higher.^24^ In particular, our analyses highlight the importance of triacylglycerol lipases, which are essential for hydrolysis of lipids into long-chain fatty acids and their subsequent assimilation and intracellular storage. The pronounced expression of this particular enzyme group suggests the possibility to enrich for lipid accumulating organisms (LAOs) in BWWT plants through lipase supplementation and environmental biocatalysis. Enhancing the growth of LAOs through such a strategy would result in the availability of excess amounts of lipid-rich biomass at the air–water interface of anoxic tanks and this could, for example, be transesterified to biodiesel, thereby allowing recovery of a significant fraction of the chemical energy contained within wastewater.^28,29^

The topological analysis of the OMMC-wide metabolic networks confirms the metabolic similarity of both autumn and winter communities, with a high centrality of central carbon metabolism. The measure of betweenness centrality demonstrates seasonal variability in fatty acid metabolism, which is more enriched in the sampled winter OMMC. The identification of genes encoding key functionalities involved the detailed analysis of topological features within the reconstructed community-wide metabolic networks as well as an assessment of relative gene expression by enzyme-coding genes. This analysis highlighted genes such as AMO, expressed by *Nitrosomonas* spp., and an acyl-[acyl-carrier protein] desaturase, expressed by *Rhodococcus* spp., as fulfilling key functions in OMMCs.

The developed framework allows the integration of structural and functional measurements through contextualisation in reconstructed community-wide metabolic networks to result in the identification of genes encoding key functionalities, which can in turn be linked to functionally important community members. These potential ‘keystone genes’ could ultimately serve as driver nodes^31^ for controlling such complex microbial ecosystems. Therefore, the application of our methodological framework to other microbial communities for the identification of keystone species may allow community-wide control strategies to be formulated where other community-wide phenotypic outcomes may be desirable, e.g., in the human gastrointestinal tract. *In silico* analysis results presented in this paper were obtained using the high performance computing facilities of the University of Luxembourg^51^.
