# Supplementary material for: Comparative integrated omics: identification of key functionalities in microbial community-wide metabolic networks
Source: NPJ Biofilms Microbiomes. 2015 Jun 17;1:15007–. doi: 10.1038/npjbiofilms.2015.7 (PMC5515219; doi:10.1038/npjbiofilms.2015.7)
Supplement: Supplementary Information [file npjbiofilms20157-s1.pdf]

SUPPLEMENTARY INFORMATION

Comparative integrated omics: identification of key functionalities in microbial community-wide metabolic networks

Hugo Roume[a,1*], Anna Heintz-Buschart[a*], Emilie EL Muller[a], Patrick May[a], Venkata P Satagopam[a], Cédric C Laczny[a], Shaman Narayanasamy[a], Laura A Lebrun[a], Michael R Hoopmann[b], James M Schupp[c], John D Gillece[c], Nathan D Hicks[c], David M Engelthaler[c], Thomas Sauter[d], Paul S Keim[c], Robert L Moritz[b], and Paul Wilmes[a,2]

[a]*Luxembourg Centre for Systems Biomedicine, University of Luxembourg, Esch-sur-Alzette, Luxembourg.*

[b]*Institute for Systems Biology, Seattle, WA, USA.*

[c]*The Translational Genomic Research Institute-North, Flagstaff, AZ, USA.*

[d]*Life Science Research Unit, University of Luxembourg, Luxembourg, Luxembourg.*

---

[1]Current address: Laboratory of Microbial Ecology and Technology, Ghent University, Belgium

[2]Corresponding author: P Wilmes, Luxembourg Centre for Systems Biomedicine, University of Luxembourg, L-4362 Esch-sur-Alzette, Luxembourg. E-mail: paul.wilmes@uni.lu.

* These authors contributed equally to this work.

Supplementary Materials and Methods

Biomolecular extraction and quality assessment

For each sampling date, three 200 mg sub-samples derived from one OMMC islet (defined, herein[1], as technical replicates) were used for biomolecular extraction. Each of the individual biomolecular fractions isolated from the technical replicates were combined into a pool in order to yield sufficient biomolecular quantities for subsequent high-throughput analysis and to reduce the influence of fine-scale within-sample heterogeneity (as demonstrated by Roume *et al.*[2]).

For the quality assessment of the isolated genomic DNA, fractions were separated by electrophoresis on a 1 % agarose gel containing 4 ‰ ethidium bromide (PlusOne Ethidium Bromide, GE Healthcare). For size estimation, the MassRuler DNA ladder mix (Fermentas) was loaded onto the gels. Agarose gels were visualized on an InGenius gel imaging and analysis system (Syngene, Cambridge, UK; as described in Roume *et al.*[1]). The DNA pool sample was snap-frozen in liquid nitrogen in the elution buffer and stored at -80 °C until library preparation and sequencing.

RNA quality assessment and quantification was carried out using an Agilent 2100 Bioanalyzer (Agilent Technologies, Diegem, Belgium; as described in Roume *et al.*[1]).

The quality of protein extracts was assessed following 1D-SDS-PAGE separation.

Preparation of RNA for shipment and sequencing

The volume of the RNA pool sample was adjusted to 180 µl using RNase free-water. A mixture of 18 µl of a 3 M (w/v) sodium acetate solution, 2 µl of a glycogen solution

(10 mg/µl) and 600 µl of ice-cold 96 % (v/v) ethanol was added to the RNA solution. The RNA solution was gently mixed by inversion and precipitated at -20 °C for at least 1 h. Following centrifugation at 10 000 *x* g for 30 min at 4 °C, the RNA pellet was washed three times with ice-cold 70 % (v/v) ethanol. The pellet was then air dried for 5 min at room temperature overlaid with 100 µl of Ambion® RNAlater (Life Technologies, Gent, Belgium) and placed at -20 °C until shipment. For reclamation of the RNA, the solution was centrifuged at 14 000 *x* g for 10 min at 8 °C. After removal of the supernatant, the pellet was washed three times with 100 µl of 80 % (v/v) ethanol, followed by 25 000 *x* g centrifugation for 3 min at 8 °C. The pellet was then air dried until all visible ethanol had evaporated. The dried RNA pellet was then re-suspended in 1 mM sodium citrate buffer solution (pH 6.4).

RNA-Sequencing

In order to enrich the RNA fraction in mRNA, the rRNA was subtracted from the total RNA fraction using the Ribo-Zero rRNA Removal Kit (Meta-Bacteria; Epicentre, Madison, WI, USA). This was followed by cDNA synthesis and amplification using the ScriptSeq™ v2 RNA-Seq library preparation kit (Epicentre).

rRNA removal

The procedure consisted of an initial washing and re-suspension of the Ribo-Zero microspheres with dedicated solutions. Following treatment of the total RNA sample with Ribo-Zero removal solution, the RNA sample solution was added to the re-suspended Ribo-Zero microspheres and selected by hybridisation. The RNA-microsphere solution was then removed and the rRNA-depleted sample was purified by ethanol precipitation.

Library preparation

The cDNA synthesis and amplification were performed using the ScriptSeq^{TM} v2 RNA-Seq library preparation kit (Epicentre). The procedure consisted of initial fragmentation of the RNA, followed by the annealing of the cDNA synthesis primer. Briefly, addition of 4 µl cDNA Synthesis Master Mix to the fragmented RNA solution was followed by incubation first at 25 °C for 5 min and then 42 °C for 20 min. cDNA Synthesis Master Mix was prepared by combining 3 µl cDNA Synthesis PreMix (ScriptSeq v2 RNA-Seq library preparation kit), 0.5 µl of DTT (100 mM) and 0.5 µl of StartScript Reverse Transcriptase solution (Epicentre). After cooling to 37 °C, 1 µl of finishing solution (Epicentre) was added to the cDNA synthesis solution and incubated at 37 °C for 10 min, before an incubation at 95 °C for 3 min. 8 µl of terminal tagging master mix was then added to each solution and incubated at 25 °C for a further 15 min, following incubation at 95 °C for 3 min. Terminal tagging master mix was prepared using 7.5 µl of terminal tagging premix (Epicentre) and 0.5 µl of DNA polymerase. The 3'-terminal tagged cDNA was then purified using the AMPure XP system (Beckman Coulter, Brea, CA, USA). The purified cDNA strand was then amplified by PCR, resulting in the generation of the second strand of cDNA, addition of the Illumina adaptor sequences and incorporation of specific barcodes, as well as amplification. Finally, the RNA-Seq library was purified using AMPure XP system (Beckman Coulter). The size distribution of the RNA-Seq library was assessed using an Agilent 2100 Bioanalyzer.

DNA/cDNA sequencing

DNA/cDNA was prepared according to the modified instructions from The Wellcome Trust Sanger Institute[3]. The metagenomic sequencing protocol used a 96-well library preparation and the molecular barcoding method for Illumina library construction. The barcodes were

designed using Hamming codes, which allow single nucleotide sequencing errors to be corrected and single indels (insertions/deletions) to be detected without ambiguity. Several optimisations were employed in the indexing protocol. The tags used were 8 bp long, which allowed the design of larger number of barcodes with error-correcting capability. The bar codes were introduced in a regular PCR, which simplified the PCR step and allowed for use of as few as six cycles. Before pooling, the relative concentration of each sample library was measured by quantitative PCR (qPCR), which then allowed the accurate pooling of libraries together and improved the uniformity of their representation.

DNA fragmentation

1-5 µg of DNA was used for the fragmentation step, as determined following gel analysis, and re-suspended in 75 µl of 10 mM Tris-HCl, pH 8.5. The sample was then sheared for 150 s in 100 µl Covaris microtubes (Woburn, MA, USA). The following programme was used: duty cycle 20 %, intensity 5, cycle burst 200, power 37 W, temperature 7 °C and mode 'Freq sweeping'. The sheared DNA samples were transferred into a MicroAmp optical 96-well plate (Life technologies, Grand Island, NY, USA). Six samples, chosen at random, were run on an Agilent Bioanalyzer DNA 1000 chip to check the quality of the fragmentation. A 150-250 bp smear was detected and 70-90 % of the initial DNA amount was recovered.

Size selection

A large fragment plate was prepared by dispensing 150 µl of beads into wells of a round-bottom Costar plate (Corning, Glendale, AZ, USA) for each sample. Additionally, a small-fragment plate was prepared by dispensing 60 µl of beads into wells of a separated Costar plate for every sample. The large-fragment Costar plate was placed on a magnetic stand,

allowing the collection of beads. 45 µl of 10 mM Tris-HCl buffer were removed from the large-fragment wells, leaving all beads in the well. The plate was removed from the magnetic stand and 190 µl of sample from sonication tubes was added to the large fragment wells on the Costar plate. Following 8 min of incubation at room temperature, using two magnetic stands, the large-fragment Costar plate was placed on a magnetic stand to collect beads. Following 5 min incubation at room temperature, 58 µl of bead buffer were removed from the small fragment wells, leaving behind beads and approximately 2 µl of bead buffer. Small-fragment wells were then removed from the magnetic stand. 300 µl of supernatant were transferred from large-fragment wells into small-fragment wells. After transfer, the large-fragment plate was discarded. Samples were then incubated in small-fragment wells for 5 min and the plate was placed on a magnetic plate to collect beads. 300 µl of supernatant were removed and replaced by 300 µl of 80 % ethanol without disturbing beads. Following 30 s of incubation, ethanol was removed. This last step was repeated two times. Following ethanol removal by air-drying for 5 min, the small fragment plate was removed from the magnetic stand and 45 µl of pre-warmed 10 mM Tris-HCl (pH 8.5) were dispensed into the wells. After re-suspension of beads by vigorous mixing, the solution was incubated for 2 min and placed on a magnetic plate to collect beads. Following a further 3 min incubation, 42.5 µl of supernatant containing the size-selected products were transferred to a new plate.

All enzymes and reaction buffers used for the end-repair, dA-tailing, ligation and PCR amplification were provided from the KAPA Library Preparation Kits with Standard PCR Library Amplification/Illumina series (KapaBiosystems, Woburn, MA, USA).

End-repair

To the supernatant containing the sheared DNA, 10 µl of end-repair buffer and 5 µl of end-repair enzyme mix were added. 100 µl of this solution were added to each well. The plate was then covered, vortexed briefly and spun down before being incubated for 30 min at 20 °C in a thermocycler. The samples were then cleaned using Agencourt AMPure SPRI beads (Beckman Coulter). 90 µl of SPRI beads were added into each well, the plate was covered and vortexed for 30 s. The reaction plate was placed on the magnetic SPIRPlate for 10 min and beads separated from the solution. Following incubation for 10 min, the completely clear solution was discarded. 200 µl of 70 % (v/v) ethanol were added to each well and incubated for 30 s at room temperature. The ethanol washes were repeated two times. The reaction plate was dried for 15 min in a thermocycler and each sample was eluted with 43.5 µl of 10 mM Tris buffer (pH 8.0).

A-tailing

To 30 µl of end-repaired DNA, 5 µl of 10x A-tailing buffer, 3 µl of A-tailing enzyme and 12 µl of water were added. 50 µl of A-tailing master mix were added to each well. The plate was then covered, vortexed briefly and spun down before being incubated for 30 min at 30 °C in a thermocycler. The sample was then cleaned up using the Agencourt AMPure SPRI bead method with 90 µl of SPRI beads placed in each well. Each sample was then eluted with 36 µl of 10 mM Tris buffer (pH 8.0). The same six samples, randomly chosen for the DNA fragmentation step, were run on an Agilent Bioanalyzer DNA 1000 chip and the average sample concentration was calculated using the "integrated peak" function.

Adapter preparation and ligation

To 30 µl of A-tailed DNA, 10 µl of 5x ligation buffer, 5 µl of DNA ligase and 5 µl of DNA adaptor (30 µM) were added. 50 µl of this reaction mixture, were added to each well. The plate was then incubated at 20 °C (room temperature) for 15 min. The sample was cleaned up using Agencourt AMPure SPRI beads by addition of 40 µl of SPRI beads to each well. Each sample was then eluted with 30 µl of 10 mM Tris buffer (pH 8.0) / 0.05 % (v/v) Tween 20. The same six samples were randomly chosen for the DNA fragmentation step and run on an Agilent DNA 1000 chip to check the success of the ligation; the smear obtained had an average molecular size of 50 to 200 bp larger than before ligation.

PCR amplification

Library amplification was carried out according to a modified reaction setup as defined in the KAPA Library Preparation kit instructions (Illumina). 25 µl of 2x Kapa HIFI Hotstart Mix were spiked with 1 M betaine, aiding in amplification of high-GC-content regions and reducing biases. 1 µl of the supplied PCR primers were added to each tube and a sufficient quantity of water was added to reach 50 µl of solution volume per PCR reaction. The tubes were then transferred into a PCR thermocycler and run with the recommended KAPA library preparation cycling programme. The samples were then cleaned using the SPRI beads method with 40 µl of SPRI beads. Each sample was eluted using 50 µl of 10 mM Tris-HCl / 0.05 % (v/v) Tween 20. The same six samples as those randomly chosen at the DNA fragmentation step, were run on an Agilent Bioanalyzer DNA 1000 chip to check the success of the indexing enrichment PCR.

Final library quantification by qPCR

The KAPA Library Quant kit (KapaBiosystems) for final library quantification was used (Illumina). Briefly, after an initial 1:1 000 dilution in 10 mM Tris-HCl, pH 8.0 + 0.05 % (v/v) Tween 20, the 2x KAPA SYBR FAST qPCR master mix was used to amplify the DNA library with six other standards on an ABI 7900 thermocycler. The qPCR step was conducted using the following cycling conditions: 95 °C for 5 min followed by 35 cycles at 95 °C for 30 s and at 60 °C for 45 s. The concentration of the library was established using the standards according to the manufacturer's instructions. Both the standards and the libraries with unknown concentration were run in triplicate. Prior to sequencing, the libraries were diluted to the required concentration (e.g. 4.5 pM) by following the Illumina cluster generation protocol.

Sequence assembly

For each of the two sampling dates, the raw paired-end 100 nt read metagenomic and metatranscriptomic sequences were processed separately first using the *PAired-eND Assembler*[4] (*PANDAseq*, **Supplementary Figure 1**, step 1) to assemble overlapping read pairs. *PANDAseq* was run with a score threshold of 0.9 and 25 nt minimum overlap requirement to determine the location of the amplification primers, identify the optimal overlap between reads, correct sequencing errors and check length and base quality. The reads selected by the *PANDAseq* assembler were extracted from the raw sequence files using in-house Perl scripts. The remaining non-redundant paired-end reads were trimmed using the *trim-fastq.pl* script from the *PoPoolation* package[5] using a quality-threshold of 20 (1 % probability of miscall) and a minimum length of 40 nt resulting in two quality trimmed read sets, one including still paired-end and one containing only single-end reads, where the other

read pair was discarded during quality trimming. Metagenome and metatranscriptome FASTQ files were then combined into a combined FASTQ file. All *PANDAseq* and single-end reads were then combined into a single FASTQ file. Paired-end and single-end reads were made non-redundant using *CD-HIT-dup*[6] (**Supplementary Figure 1**, step 2). None-redundant reads were then used as input for the *MOCAT* assembly pipeline[7] (**Supplementary Figure 1**, step 3), using default parameters. The assembled contigs were filtered with minimum length threshold of 150 nt. To enhance the final assembly by reads that were assembled by *PANDAseq*, but not used by the *MOCAT* assembly pipeline, all *PANDAseq* reads were mapped onto the *MOCAT* contigs using *SOAPaligner*[8], with the following parameter settings: -r 2 -M 4 -l 30 -v 10 -p 8 (**Supplementary Figure 1**, step 4). The unmapped *PANDAseq* assembled reads with a minimum length of 150 bp, were extracted and added to the contigs. The final contig files were made non-redundant using *CD-HIT*[6] (-c 1.0) by clustering identical sequences.

Extraction and classification of reads mapping to rRNA genes in the metagenomic data

EMIRGE[9] was run using metagenomic reads quality filtered to minimum average QV = 30 and a minimum of 40 bp with the *trim-fastq.pl* script from the *PoPoolation* package[5]. The reference database used was the truncated (non-redundant) small ribosomal subunit SILVA[10] database release 111. The consensus sequences at 100 iterations were extracted and named based on their identification in the reference database, without imposing any normalized posterior probability.

Generation of additional metagenomic data for contig extension and analysis

To obtain an additional high-depth metagenomic dataset, a total of four additional floating

sludge samples islets were collected on 23rd February 2011, each representing a biological replicate. Biomolecular extraction was performed on 200 mg of starting material as for the other two dates, using the protocol described by Roume *et al.*[1]. The resulting DNA fractions were sequenced under sample names I, II, III and IV using the sequencing protocol described above. Four technical replicates from sample I were generated, resulting in four libraries I-1, I-2, I-3 and I-4. While the remaining biological replicates II, III and IV were sequenced once each, generating a total of 12.6 gigabases metagenomic sequence. The reads were then assembled using *AMOS*[11] and *MetaVelvet*[12].

Gene annotation

Non-redundant contig files were split into two distinct files, one file with contigs of a length below 500 bp and another file with contigs lengths above or equal to 500 bp. The contigs with lengths below 500 bp were annotated using *FragGeneScan*[13] (**Supplementary Figure 1**, step 5), using settings for short sequence reads with sequencing error (-complete 0 –train illumine_5). The contigs with a length equal or above 500 bp were annotated with *Prodigal gene finder*[14] (v2.60, **Supplementary Figure 1**, step 5) using the *MOCAT* gene prediction processing steps. The resulting amino acids sequence files were then combined into a single file and made non-redundant using *CD-HIT* with a sequence identity threshold of 1.0 and a description string length within the cluster file of 5 000 (-c 1.0 –d 5000; **Supplementary Figure 1**, step 6).

All sequences were mapped to the KEGG database version 64.0 using *BLAT*[15] and sequences were annotated with KOs (KEGG orthologous groups; **Supplementary Figure 1**, step 7).

Pre-processing of protein fraction for high-throughput analysis

Following 1D-SDS-PAGE electrophoresis and staining with Imperial protein stain (Thermo Scientific, Erembodegem, Belgium; as described in Roume *et al.*[1]), the protein gel was conserved at 4 °C in the dark and under vacuum in sealing foil D0316L-20 (DOMO ELEKTRO, Herentals, Belgium). Prior to further analysis, entire lanes were cut into 1 mm-slices using a grid cutter (MEE-1x5, Gel Company, San Francisco, CA, USA), yielding approximately 70 slices per lane. Two 1 mm-slices were combined in a single well of a 96-well V-bottom plate with a hole introduced into the bottom using a 30 gauge lancet needle (Becton Dickinson, Franklin Lakes, NJ, USA). The wells contained size 11 black hexagonal glass beads (SB3656, Fusion Beads) to prevent the gel pieces from clogging the hole. For in-gel digestion, an automated liquid handling system (Tecan EVO, Männedorf, Switzerland) was used for reduction, alkylation, tryptic digestion and peptide extraction from the gel pieces. After extraction, the peptide solution was dried and reconstituted in 20 µl of a solution of 0.1 % (v/v) [trifluoroacetic acid (TFA, 5 %; Sigma) / acetonitrile (ACN, 95 %; BioSolve, Valkenwaard, Netherlands)] in MilliQ H_2O in a round bottom polypropylene 96-well plate (Greiner Bio-One, Monroe, NC, USA) and placed into an Eksigent Nano 2D plus system autosampler (ABSciex, Framingham, MA, USA) for analysis.

Liquid chromatography

Peptides obtained from the 1 mm-gel bands were separated using an Eksigent Nano 2D LC plus system employing splitless nanoflow. Reverse phase high performance liquid chromatography (RP-HPLC) and separation columns were prepared in-house by packing a Kasil fritted capillary [360 µm outer diameter (OD), 75 µm inner diameter (ID)] with a 1 cm bed of ReproSil Pur C18-AQ 3 µm 120 Å stationary phase (Dr. Maisch GmbH, Ammerbuch,

Germany) for the sample trap and desalting column. A Kasil fritted capillary (360 μm OD, 75 μm ID) was packed with a 15 cm bed of the same stationary phase as the separation column and this was connected to a PicoTip emmiter (360 μm OD x 20 μm ID, Tip 10 μm, FS360-20-10-N-20) for nano-electrospray ionisation. For each LC run, the sample was injected for 10 minutes at 2.5 μl/min with loading buffer (2 % v/v acetonitrile and 0.1 % v/v formic acid). The sample was separated by a linear gradient changing from 98 % solvent A (0.1 % v/v formic acid in water) and 2 % solvent B (0.1 % v/v formic acid in acetonitrile) to 40 % A and 60 % B in 60 min at 0.3 μl/min.

Mass spectrometry

Following LC separation, the peptides were analysed on a LTQ-Velos Orbitrap (Thermo-Fisher, San Jose, CA, USA). MS1 data were collected over the range of 300 – 2 000 *m/z* in the Orbitrap at a resolution of 30 000. Fourier-transform mass spectrometry (*FTMS*) preview scan and predictive automatic gain control (pAGC) were enabled. The full scan *FTMS* target ion volume was 1 x 10^6 with a maximum fill time of 500 ms. MS2 data were collected in the LTQ-Velos with a target ion volume of 1 x 10^4 and a maximum fill time of 100 ms. The 10 most intense peaks were selected (within a window of 2.0 Da) for higher-energy collisional dissociation at 15 000 resolution in the Orbitrap. Dynamic exclusion was enabled in order to exclude an observed precursor for 180 s after two observations. The dynamic exclusion list size was set at the maximum 500 and the exclusion width was set at ±5 ppm based on precursor mass. Monoisotopic precursor selection and charge state rejection were enabled to reject precursors with $z = +1$ or unassigned charge state.

Protein identification

For MS analysis, Thermo .RAW files were converted to mzXML format using *MSConvert* (*ProteoWizard*[16]) and searched with *X!Tandem*[17] version 2011.12.01.1. Spectra were searched against the metagenomic and metatranscriptomic data, common lab protein contaminants, and decoys. Redundancy was removed from these three data sets using *BlastClust*. The contaminant database was a modified version of the common Repository of Adventitious Proteins (cRAP, www.thegpm.org/crap) with the Sigma Universal Standard Proteins removed and human angiotensin II and [Glu-1] fibrinopeptide B (MS test peptides) added, for a total of 66 entries. Decoys were generated with *Mimic* (www.kaell.org), which randomly shuffles peptide sequences between tryptic residues, but retains peptide sequence homology in decoy entries.

Search criteria used for *X!Tandem* included a precursor mass tolerance of 15 ppm and a fragment mass tolerance of 15 ppm for higher-energy collisional dissociation spectra. Peptides were assumed to be semi-tryptic (cleavage after K or R except when followed by P), but semi-tryptic peptides with up to 2 missed cleavages were allowed. The search parameters included a static modification of +57.021464 Da at C for carbamidomethylation by iodoacetamide and potential modifications of + 15.994915 Da at M for oxidation, -17.026549 Da at N-terminal Q for deamidation, and -18.010565 Da at N-terminal E for loss of water from formation of pyro-Glu. Additionally, -17.026549 Da at the N-terminal carbamidomethylated C for deamidation from formation of S-carbamoylmethylcysteine and N-terminal acetylation were searched. Peptide spectrum matches (*PSMs*) obtained from *X!Tandem* were validated using the *Trans Proteomic Pipeline*[18] version 4.6 Rev.1. The *PSMs* were analysed with *PeptideProphet*[19] to assign each *PSM* a probability of being correct. Accurate mass binning was employed to promote *PSMs* whose theoretical mass closely matched the observed mass of the precursor ion, and to correct for any systematic mass

errors. Decoys and the non-parametric model option were used to improve *PSM* scoring. Protein identifications were inferred with *ProteinProphet*. The ProteinProphet scores were then analysed in *iProphet*[20], which combines results from multiple fractions and multiple database searches (although here, only *X!Tandem* was used) and assigns a probability for each unique protein and its corresponding peptide sequences. The false discovery rate for a given *iProphet* probability was calculated using the number of decoy protein inferences at that probability. Only proteins identified at *iProphet* probabilities corresponding to a false discovery rate (FDR) less than 1.0 % were further considered.

KO annotations and protein quantification

The corresponding sequences of the identified proteins were collected in FASTA format from the non-redundant single peptides/protein sequences database previously generated from the combined metagenome and metatranscriptome assemblies. Relative protein quantitation was performed using the normalized spectral index (*NSI*) measure using an in-house software tool called *NSICalc* as previously described[21]. The amino acid sequences of identified proteins were then mapped to the KO library (KEGG database version 64.0) using *BLAT*[15] (e-value$<10^{-5}$, %identity>50, score>50). From the resulting list of KOs, the frequency of each KO was determined at the protein level, using an in-house developed Perl script.

Gene copy and transcript abundances

To account for differences in read depth and sampling, the number of raw sequence reads from autumn and winter metagenome and metatranscriptome libraries were equilibrated by randomly selecting reads in the larger libraries from the autumn sample to mirror the number of reads in the smaller library (winter) using an in-house Perl script based on the 'shuffle'

method from the 'List::Util' CPAN package. This resulted in 14 546 374 reads and 16 443 761 reads being used from the metagenomic and metatranscriptomic libraries, respectively. The four balanced raw read sequence libraries were then mapped separately to the combined assembly for both sampling dates using *SOAPaligner*[8] with the following parameter settings: -r 2 -M 4 -l 30 -v 10 -p 8.

For each library, reads were mapped to genes and counted, except for reads mapping to multiple genes, for which weighted proportions were used. Next, to obtain the abundances of genes and transcripts, read counts were normalized by the length of the respective gene sequences[22], to obtain normalized gene copy abundances and normalized transcript abundances, respectively. Normalized gene copy abundances per KO were obtained by calculating the sum of normalized gene copy abundances from all genes belonging to the same KO group. Similarly, the KO-wise transcript abundances were calculated as the sum of normalized transcript abundances over all genes within the same KO.

Relative gene expression

Relative expression of KOs was determined by dividing the normalized transcript abundance of each KO by the inferred gene copy abundance of the same KO[23].

The relative expression of a KO is greatly dependent on the normalized gene copy abundance, if this value is close to zero. Similarly, KOs with normalized gene copy abundances close to zero are prone to be falsely identified as highly expressed due to their very low gene copy abundances. Therefore, highly expressed KOs were selected based on normalized gene copy and transcript abundances, in addition to relative expression. KOs were considered highly expressed, if their relative expression was above the 90th percentile. In addition, to avoid false positive identification of highly expressed KOs, the normalized

transcript abundances of highly expressed KOs had to be above the 3rd quartile of the normalized transcript abundances of all KOs or normalized gene copy abundances had to be above an empirically determined threshold. This threshold was determined by sorting KOs by their normalized gene copy abundances and applying a sliding window approach to determine the lowest normalized gene copy abundance with an average relative expression robustly within the interquartile range (see **Supplementary Figure 2**).

Sensitivity of gene expression analysis to imposed cut-offs

Results of the analysis of KOs exhibiting high relative expression are dependent on the quantile of KOs considered highly expressed (we considered KOs with a relative expression above the 90th percentile), as well as the lower cut-off which was set for gene copy abundances to avoid false positive identification of KOs exhibiting very low gene copy abundances and only slightly higher transcript abundances as highly expressed. Therefore, we first analysed, if the numbers and identities of KOs identified based on their relative expression were robust to different levels of noise added to the data. In addition, we analysed whether the conclusions would also be robust to small to moderate changes in the selected cut-off values.

To address the first point (robustness to noise), we changed the gene copy and transcript abundances by a random number following a uniform distribution within different limits. The lowest of these limits corresponded to +/- 1 read mapped per kilobase of metagenomic sequence and the highest to +/- 50 reads mapped per kilobase. We then analysed the numbers and identities of the highly expressed genes in 100 repetitions of each test, given the chosen cut-offs and the identities of enriched pathways within the selected sets of KOs. We compared the results to the same analysis carried out using a single numerical cut-off for

relative expression (minimal relative transcript abundance = 10 times relative gene copy abundance).

To address the second point (robustness to changes in cut-offs), we changed each cut-off within small to moderate limits. For the inclusion of KOs irrespective of their gene copy abundances, cut-offs at steps between the 55th to 95th percentile of transcript abundances were used. For the exclusion of KOs with low gene copy abundances, different cut-offs of robust relative expression were set between the 20th and 95th percentile. We then analysed the numbers and identities of the KOs found to be highly expressed, as well as the pathways enriched in these KOs.

Comparison of the metagenomic dataset with the metatranscriptomic and metaproteomic datasets

The congruency of metagenomic and metatranscriptomic datasets was determined by calculating the proportion of KOs with at least one gene having at least 10 metagenomic reads per kilobase mapping to it and also at least one gene resulting in the mapping of 10 metatranscriptomic reads per kilobase. The congruency of the metagenomic and metaproteomic datasets was calculated analogously as the proportion of KOs with at least one gene mapped by at least 10 metagenomic reads per kilobase that also had at least one gene identified at the protein level.

Analysis of pathway membership

Assignment of KOs to KEGG pathways was done by using the KO to pathway link (http://rest.kegg.jp/link/Ko/pathway) in the KEGG database version 67.1. Enrichment of KOs

in specific pathways was tested using a hypergeometric test and p-values were adjusted using FDR-control[24]. Test results with adjusted p-values below 0.05 were considered significant.

Community-wide metabolic network reconstructions

The KO to R (reaction) link (http://rest.kegg.jp/link/Ko/reaction) was used to associate each individual KO to the corresponding reactions following the R to RP (reaction pair) link (http://rest.kegg.jp/link/reaction/RP), thereby, associating individual reactions to their corresponding main reaction pairs. The RPAIR annotation was specifically chosen to ignore unspecific compounds of reactions (water, energy carriers and cofactors), thereby, only taking into account the main compounds of a reaction. The reaction pairs (RP) were then further selected by using only RPAIRs with assigned reaction classes (http://rest.kegg.jp/link/rn/rc). Finally, the reaction pair - compounds link (http://rest.kegg.jp/list/RP) was used to associate individual RPs to corresponding pair(s) of compounds. As some KOs have identical compounds (e.g. subunits of the same enzyme or enzymes that catalyse each other's reverse reaction), KOs with identical compounds (as annotated in the KEGG database version 67.1) were grouped. A KO network graph was built by using all KOs as nodes. Edges between two KOs were introduced if a product metabolite of one KO was found as a substrate metabolite of the other KO. Multiple edges between the same KOs were reduced into a single edge. For topological analysis of the reconstructed metabolic network, nodes sharing the exact same edges were regrouped to be represented as a single node. Multiple edges connecting the same nodes were likewise combined into a single edge. The undirected network graph was visualized and analysed using *Cytoscape*[25], employing a spring embedded layout. Singleton nodes not connected to any other node were removed.

Calculation of gene copy and transcript number and relative expression of regrouped nodes

Gene copy numbers and transcript numbers of nodes which represented several KOs due to their sharing of the same edges were calculated by summing all normalized gene copy and transcript numbers, respectively. Relative expression of a node was calculated by dividing the node-wise sum of normalized transcript abundances by the node-wise sum of normalized gene copy abundances, thereby levelling relative expression of the regrouped KOs.

Identification of choke points

Choke points as defined by Rahman and Schomburg[26] are enzymes which consume or produce unique metabolites and possess a high *load score* in the metabolic network reconstruction. To assess whether a node within the metabolic network reconstructions could qualify as choke point, the number of edges representing every metabolite was counted, yielding the *number of occurrence*. In the cases where an edge represented more than one metabolite, the lowest *number of occurrence* was assigned to this edge. Every node was also assigned the lowest *number of occurrence* of all its edges. Nodes with an assigned *number of occurrence* of 1 were considered potential choke points. The *number of occurrence* for every node is listed and potential choke points are highlighted in **Supplementary Dataset 7.**

Weighted load score

Weighting of compounds in a bi-partite RPAIR-based metabolic network reconstruction has been shown to increase pathfinding accuracy[27]. The *number of occurrence* described above was assigned as edge weight within the metabolic network reconstructions. A weighted betweenness centrality was calculated using the R-package *igraph*[28]. Alternative weighted *load scores* were calculated for each node from the weighted betweenness centralities and the

degree as defined in the manuscript, and potential key functionalities were determined using this measure and expression as described in the main manuscript. The resulting weighted *load scores* and the identities of the key nodes were compared to the unweighted results discussed in the manuscript.

Matching of genes to Candidatus Microthrix parvicella Bio17 genome

Amino acid sequences were aligned using *BLAT*[15] with cut-offs chosen as follows: e-value < 10^{-5}, %-identity > 50, score > 50.

Alignment of contigs encoding key functionalities

Contigs were selected based on the following criteria: (1) they encoded a gene annotated with a KO representing a key functionality, and (2) the expression of this gene was corroborated by at least one mapped metatranscriptomic read. Selected contigs were aligned to the NCBI non-redundant nucleotide database using *BLASTn* with default parameters[29]. The best hit with a query coverage above 50 % and a percentage identity above 80 % for each contig was documented. In addition, contigs containing genes annotated as K03921 were aligned to 85 isolate genomes from the same biological wastewater treatment (BWWT) plant using *BLAST* and selecting only results with percentage identity above 80 %.

Quantification of isolate sequences in combined metagenomic and metatranscriptomic assemblies

Reads from the balanced libraries (see section *Expression analysis and contextualization of omic datasets - gene copy and transcript abundances*) were mapped against the genome of

Nitrosomonas sp. Is79 (Ref. 30) and Isolate LCSB065 using *SOAPaligner*[8] with the following parameter settings: -r 2 -M 4 -l 30 -v 10 -p 8 and mapped reads were counted.

Ammonia monooxygenase (AMO) contig extension

Genes encoding subunits A and B of ammonia monooxygenase (*amoA* and *amoB*) are established phylogenetic markers[31]. However, none of the contigs from the combined autumn and winter assembly that contained an open reading frame annotated as encoding for a subunit of AMO (K10944, K10945, or K10946) harboured a complete gene. In order to recover a full gene sequence and determine the position of the *amoA* genes recovered from the combined metagenomic and metatranscriptomic data relative to other known *amoA* sequences within a phylogenetic tree, we employed a contig extension protocol in order to increase the length of the contigs via extension and merging of these contigs.

The contig extension protocol was carried out in a step-wise fashion including contig extension, gene calling and gene annotation. Contig alignment, merging and extension was performed using *minimus2* (Ref. 32) from the *AMOS suite*[33] with a minimum overlap of 60 bases at 98 % identity. The contig extension protocol was performed in three steps: i) contigs were extended by aligning contigs annotated with the same KO IDs; ii) contigs were extended by aligning contigs annotated with KOs K10944, K10945 or K10946; iii) contigs were extended using a high-depth metagenomic assembly (see section *Generation of additional metagenomic data for contig extension and analysis*) from a different sample, using the AMO contigs from the previous step as a reference. This procedure was performed, because the genes encoding the subunits of AMO are known to exist in a cluster/operon[34]. Following the run on *minimus2*, gene calling was performed on the resultant contigs using *FragGeneScan*[13] and *Prodigal*[14] using default parameters as previously used. Predicted

amino acid sequences were then merged and made non-redundant based on 100 % sequence identity using *CD-HIT*[35] and were re-annotated with KO IDs using our annotation pipeline. Gene calling and re-annotation steps were performed to ensure that the extended contigs retained their original annotation reference. The extended contigs were then used for downstream analysis in order to associate these AMO genes to a bacterial species.

AmoA phylogenetic analysis

Nearly complete amino acid sequences (201 – 274 amino acids) of AmoA and/or MmoA from representative organisms belonging to the beta-Proteobacteria, gamma-Proteobacteria and archaea were retrieved from the Refseq protein database (see **Supplementary Table 3**) and aligned with *ClustalOmega* (using default parameters). The alignment file was submitted to a phylogenetic analysis using the Phylogeny.fr customized workflow service[36] including alignment curation with *Gblocks*[37] (using default parameters), tree construction with *PhyML*[38] (bootstrap of 100), and visualization by *TreeDyn*[39].

Isolate LCSB065 isolation

Isolate LCSB065 was obtained from an OMMC biomass sample diluted by a factor of 10^4. The biomass was first cultivated on Petri dishes of wastewater-agar medium (1.5 % agar; w/v) in 800 ml filtered (0.2 µm, Sartorius, Göttingen, Germany) wastewater from the Schifflange BWWT plant. A single colony was then transferred to a Petri dish with R2A medium[40] and cultivated at 20 °C under aerobic conditions. Isolates were grown on different growth media recommended for the culture of bacteria from water and wastewater, particularly *Microthrix parvicella*, such as R2A[40], wastewater agar medium, MSV + peptone and MSV A + B[41] or Slijkhuis A and F[42] under different growth conditions.

Nile red staining

Lipid inclusions were visualized using a protocol modified from Fowler & Greenspan[43]. A stock solution of Nile red (Sigma-Aldrich, Diegem, Belgium) in acetone (Sigma-Aldrich) was prepared at a concentration of 500 μg/ml and preserved at 4 °C protected from light. 50 μL of a working solution, containing 2.5 μl of the stock solution in 1 ml of 75 % (v/v) glycerol, were deposited onto a microscopy glass slide with heat fixed bacterial cells. After 5 min incubation, epifluoresence and bright field microscopic observations of the same fields of view were carried out on an inverted microscope (Nikon Ti) equipped with a 60 × oil immersion Nikon Apo-Plan lambda objective (1.4 N.A). Intermediate magnification 1.5 × was used in order to better resolve images. Excitation light was from a Xenon arc lamp, and the beam was passed through an Optoscan monochromator (Cairn Research, Kent, UK) with 550/20 nm selected band pass. Emitted light was reflected through a 620/60 nm bandpass filter with a 565 dichroic connected to a cooled CCD camera (QImaging, Exi Blue). All imaging data were collected and analysed using the *OptoMorph* (Cairn Research, Kent, UK) and *ImageJ*[44].

Isolate genome sequencing and genome assembly

Following DNA extraction from isolate cultures using the Power Soil DNA isolation kit (MO BIO, Carlsbad, CA), a paired-end sequencing library with a theoretical insert size of 300 bp was prepared with the AMPure XP/Size Select Buffer Protocol as previously described by Kozarewa & Turner[3], modified to allow for size-selection of fragments using the double solid phase reversible immobilisation procedure described earlier by Rodrigue *et al.*[45] and sequenced on an Illumina HiSeq with a read length of 100 bp. The resulting 854 683

paired raw reads were de-duplicated with *FastUniq*[46], and quality filtered to a minimum average QV = 30 and a minimum length of 60 bp with the *trim-fastq.pl* script from the *PoPoolation suite*[5], leading to 551 103 read pairs and 145 500 single-end reads (high quality data yield of 73 %). Two separate preliminary assemblies were obtained with *IDBA-UD*[47]; v1.1.0, with parameters –mink 30 –maxk 90 –step 5 –similar 98 --pre_correction); and *SPAdes 2.5.0*, using the hammer read error correction module which filtered for contigs shorter than 200 bp. The resulting assemblies were merged with *phrap* (minimum overlap of 50 bp). The merged assembly was manually inspected with *Consed*[48] and contigs broken where merge conflicts were detected.

The resulting contigs were uploaded to and analysed using *RAST*[49]. According to this analysis, the isolate was a *Rhodococcus* sp. Similarity of contigs to bacterial genomes (NCBI database, accessed 6th March 2014) were assessed by *BLAT*[15]. The bitscore of each hit was recorded and only contigs with a hit to *Rhodococcus* spp. with a bitscore within 60 % of the best hit's bitscore were selected for the final set. This led to the removal of 120 contigs most of which had low coverage of reads.

Filtered reads were mapped onto the assembled contigs using *BWA*[50] with default parameters. Reads mapping with mapping quality scores at or above five were used to assess contig coverage (**Supplementary Dataset 8**). The final set of contigs was submitted to *AmphoraNet* server to determine the isolate species searching for 31 phylogenetic marker genes[51], as well as to *RAST*[49] (accession number 6666666.64457) and annotated.

The COG protein profile of Isolate LCSB065 was determined as previously described by Muller *et al.*[21]. Annotation of KOs was carried out on protein predictions from *RAST* as described for metagenomic proteins.

Supplementary Results and Discussion

Sensitivity of gene expression analysis to imposed cut-offs

We found that the cut-offs imposed to avoid false-positives within the highly expressed genes and genes encoding key functionalities led to a greater robustness against noise than would be observed if simple numerical cut-offs had been chosen. This was obvious from the simulation of noise, in which the number and variance of highly expressed genes grew with the noise, when a numerical cut-off was chosen, whereas the choice of cut-offs, as defined herein, resulted in mostly stable numbers (**Supplementary Figure 3a&b**).

The identities and numbers of KOs identified as exhibiting high relative expression in our datasets were not overly sensitive to the cut-offs imposed to avoid false-positive results. The numbers of highly expressed KOs decreased by less than 20 % by the exclusion of KOs with low gene copy abundances (**Supplementary Figure 3c**). As most genes with very high transcript abundances did not have low gene copy numbers (**Figure 3**), the cut-off selected for inclusion of KOs with high transcript abundances irrespective of their gene copy numbers changed the total number of highly expressed KOs by less than 5 %. If the transcript abundance cut-off for genes with low gene copy abundances was set between the 55th and 95th percentile (our default value was the 75th percentile), an enrichment with the KOs above the 90th percentile of the highly expressed KOs of 4 or 5 out of the 5 pathways in autumn, and 5 out of the 6 pathways in winter was consistently detected (**Supplementary Figure 3d**). Only few additional pathways were enriched after variation of this cut-off **(Supplementary Figure 3e**). In particular, the findings of pathways ko00910 “Nitrogen metabolism” and ko00190 “Oxidative phosphorylation” as exhibiting overall high levels of gene expression were resilient to changes in cut-offs.

In conclusion, the described gene expression analysis is robust to noise, as well as too small to moderate changes in the chosen cut-offs.

Effect of regrouping redundant KOs into single nodes

To carry out a topological analysis of the reconstructed metabolic network, nodes and edges were rendered non-redundant, by representing multiple KOs with identical substrate and product metabolites as a single node. Due to this step, 229 and 220 nodes representing more than one KO were part of the autumn and winter metabolic network reconstructions, respectively. The calculated *load scores* were overall only mildly affected by the regrouping (Spearman correlation of *load scores* in the redundant and non-redundant autumn or winter network reconstructions: 0.98). 70 % of key functionalities identified in the non-redundant network were shared between both autumn network reconstructions (40 % for the winter network reconstructions), as reported in **Supplementary Dataset 7**. The rationale behind regrouping redundant KOs into single nodes was based on the fact that most KOs represent subunits of enzyme complexes, which do not work in parallel, but rather cooperatively in metabolizing substrates. Consequently, some of the additional nodes found in the networks with regrouped redundant KOs represent multi-subunit complexes, such as AMO, and we therefore believe that the practice of regrouping redundant KOs into single nodes is warranted.

Genomic analysis of Isolate LCSB065

Mapping of filtered reads onto Isolate LCSB065's contigs revealed a mean/standard deviation empirical sequencing insert size of 244±43 bp, and a mean read depth per mapped position (coverage) of 27±11x (median 25x).

As a first approach to analyse Isolate LCSB065's genetic potential, protein coding genes were annotated with KOs as before for the metagenomic and metatranscriptomic sequences. Of utmost interest, out of 3 373 protein coding genes that could be annotated with KOs, 420 genes (12.5 %) were annotated as KOs belonging to "Lipid metabolism", according to KEGG Orthology. This relates to rank 4 behind "Amino acid metabolism" (19.1 %), "Carbohydrate metabolism" (16.2 %) and "Xenobiotics biodegradation and metabolism" (14.0 %). Similar results were obtained from COG categories[52] and SEED subsystems categories of the predicted proteins[53]. The assembled genome also encodes genes for the synthesis and polymerisation of poly-hydroxybutyrate (PHB, **Supplementary Dataset 8** indicated in **Figure 5b)** and for the synthesis of TAGs (**Supplementary Dataset 8** indicated in **Figure 5b**), inclusions of which are visible following Nile Red staining (see **Supplementary Figure 8)**. Nine other genes beside the gene matching to the three metagenomic contigs encoding acyl-[acyl-carrier protein] desaturases are annotated as desaturases in the isolate genome.

The genomic region of the gene matching to the three metagenomic contigs encoding acyl-[acyl-carrier protein] desaturases was assessed. The gene is the first gene of a syntenous block of four genes present in *Rhodococcus jostii* RHA1, *Nocardia farcinica* IF, *Godronia bronchialis* and *Tsukamurella paurometabola* DSM 20162. This block encodes the homologous fatty acid desaturase (peg.6927), followed by a tRNA dihydrouridine synthase B, a cell envelope-associated transcriptional attenuator LytR-CpsA-Psr of the subfamily A1 and a phosphate transport system regulatory protein PhoU. The genome of Isolate LCSB065 furthermore contains six genes coding for lipases with an export signal peptide, thereby reinforcing its potential keystone role in the community (**Supplementary Dataset 8**). The genomic enrichment of genes involved in lipid metabolism suggests that the isolated *Rhodococcus* sp. may occupy a function as keystone species within the sampled OMMCs. Additional work is required to elucidate the exact role of this organismal group

within the OMMC community.

List of Supplementary Figures

Supplementary Figure 1 Overview of the assembly and annotation pipeline.

Supplementary Figure 2 Determination of lower thresholds for gene abundances for the selection of highly expressed KOs within the reconstructed community-wide metabolic networks.

Supplementary Figure 3 Results of the sensitivity analyses.

Supplementary Figure 4 Expression of KOs in metabolic pathways at the protein level.

Supplementary Figure 5 Generalized OMMC-wide metabolic network reconstructed from the combined metagenomic and metatranscriptomic data of OMMCs sampled in autumn and winter.

Supplementary Figure 6 Representation of the fatty acid metabolic pathway (ko01212).

Supplementary Figure 7 OMMC-wide metabolic networks reconstructed from metagenomic and metatranscriptomic data of OMMCs sampled in autumn and winter.

Supplementary Figure 8 Micrographs of Isolate LCSB065.

List of Supplementary Tables

**Supplementary Table 1**	Physicochemical characteristics of the wastewater at the time of sampling in the anoxic tank of the Schifflange BWWT plant.
**Supplementary Table 2**	Quantitative and qualitative characteristics of biomacromolecular fractions sequentially isolated from the OMMC samples.
**Supplementary Table 3**	Statistics of the combined assembly of the metagenomic and metatranscriptomic sequence datasets.
**Supplementary Table 4**	Ammonia-oxidizing organisms with corresponding accession numbers of *amoA* genes used for the reconstruction of the phylogenetic tree.
**Supplementary Table 5**	Numbers of metagenomic and metatranscriptomic reads from autumn and winter dates mapping to the isolate genomes of *Nitrosomonas* sp. Is79 and Isolate LCSB065.

List of Supplementary Datasets

**Supplementary Dataset 1**	Dominant genera in the autumn and winter samples determined based on reconstruction of 16S rRNA gene sequences from the metagenomic data.
**Supplementary Dataset 2**	KO gene copy (*KOGA*), transcript (*KOTA*) and protein (*NSI*) abundances in datasets from autumn (04 October 2010) and winter (25 January 2011) samples.
**Supplementary Dataset 3**	Highly expressed metabolic KOs in the (a) autumn and (b) winter sample and pathways overrepresented by metabolic KOs highly expressed in the (c) autumn or (d) winter sample.
**Supplementary Dataset 4**	Generalized microbial community-level reconstructed metabolic network reconstructed using the combined metagenomic and metatranscriptomic datasets in simple interaction format.
**Supplementary Dataset 5**	Autumn-specific reconstructed microbial community-level metabolic network in simple interaction format.
**Supplementary Dataset 6**	Winter-specific reconstructed microbial community-level metabolic network in simple interaction

format.

**Supplementary Dataset 7**	Results of topological analyses. List of all nodes in the metabolic networks reconstructed from OMMCs sampled in (a) autumn and (b) winter with topological measures and expression values. (c) KOs with betweenness centrality values different between metabolic networks reconstructed from OMMCs sampled in autumn and winter, and (d) pathways enriched in these KOs. KOs with key functionality in OMMCs sampled in (e) autumn or (f) winter including topological analysis results, gene abundances and expression, as well as protein abundances and pathway membership. (g) Results of BLAST searches of all genes encoding key functionalities against publicly available bacterial genomes.
**Supplementary Dataset 8**	Summary of characteristics of Isolate LCSB065's genome, results of the phylogenetic analysis, and coverage of Isolate LCSB065 contigs by reads from the isolate genome sequencing.

Supplementary Figures

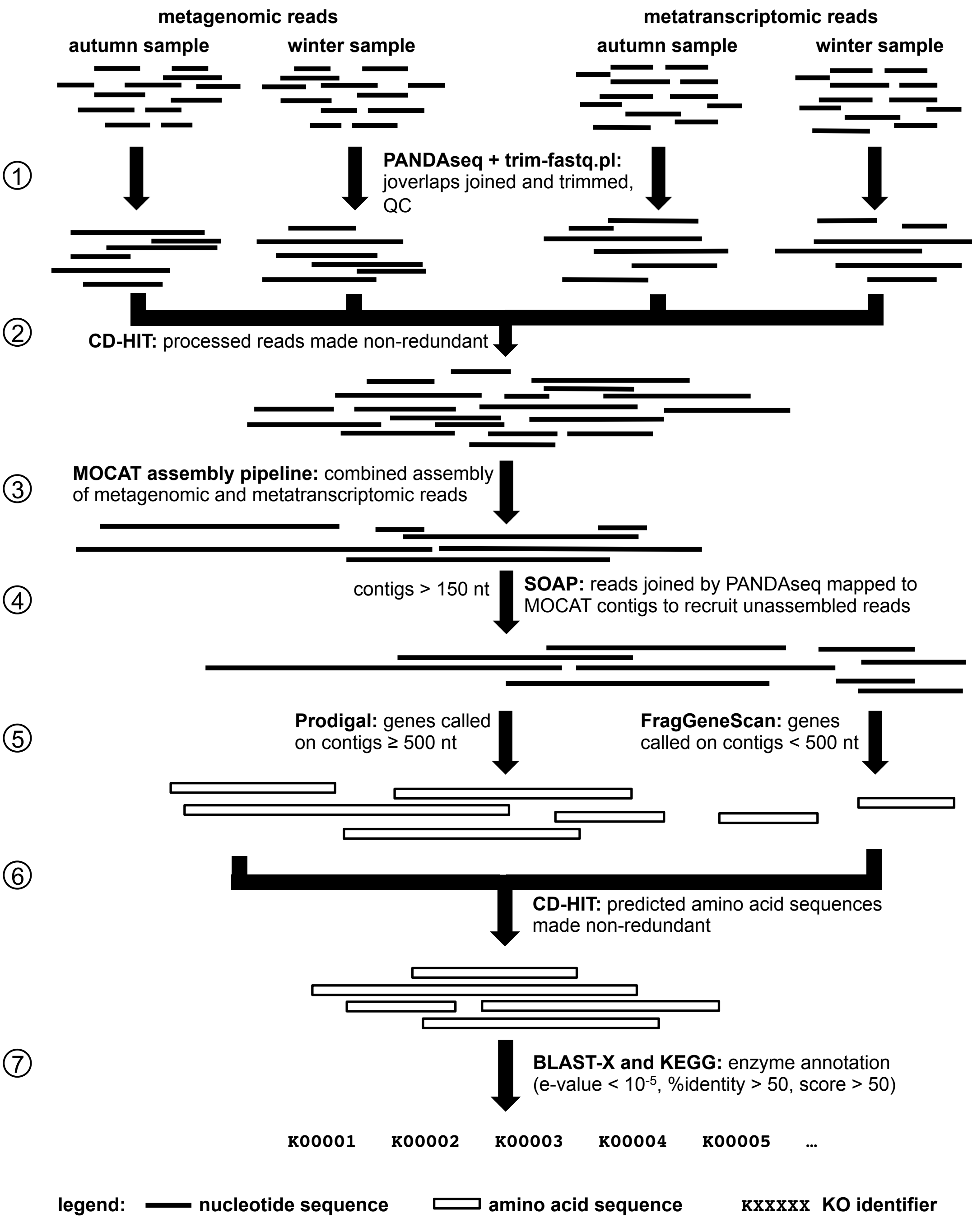

Supplementary Figure 1 Overview of the assembly and annotation pipeline. Annotated KOs were used for the subsequent metabolic network reconstructions. QC: quality control, 1-7: steps in the pipeline.

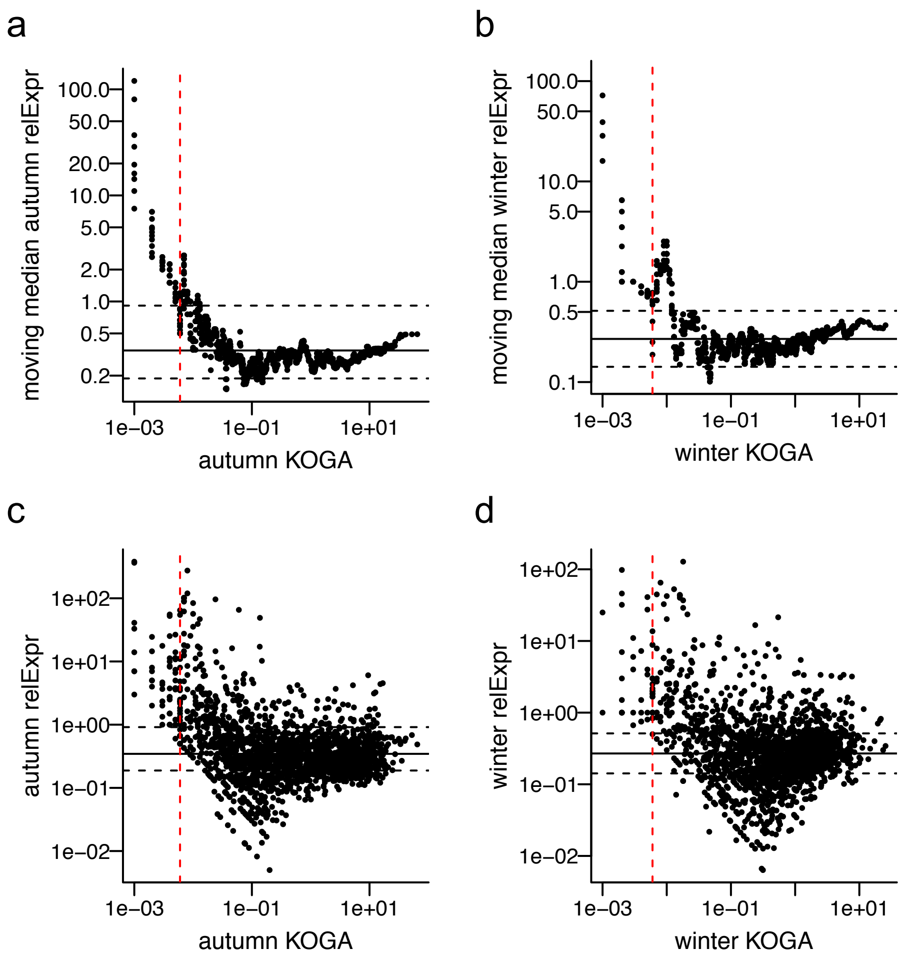

Supplementary Figure 2 Determination of lower thresholds for gene abundances for the selection of highly expressed KOs within the reconstructed community-wide metabolic networks. (**a**) and (**b**) Moving median of KO relative expression (*relExp*) versus gene copy abundance (*KOGA*) for (a) autumn and (b) winter. (**c**) and (**d**) KO relative expression (*relExp*) versus gene copy abundance (*KOGA*) for (a) autumn and (b) winter. Vertical lines indicate lowest gene abundances with robust gene expression values within the interquartile range.

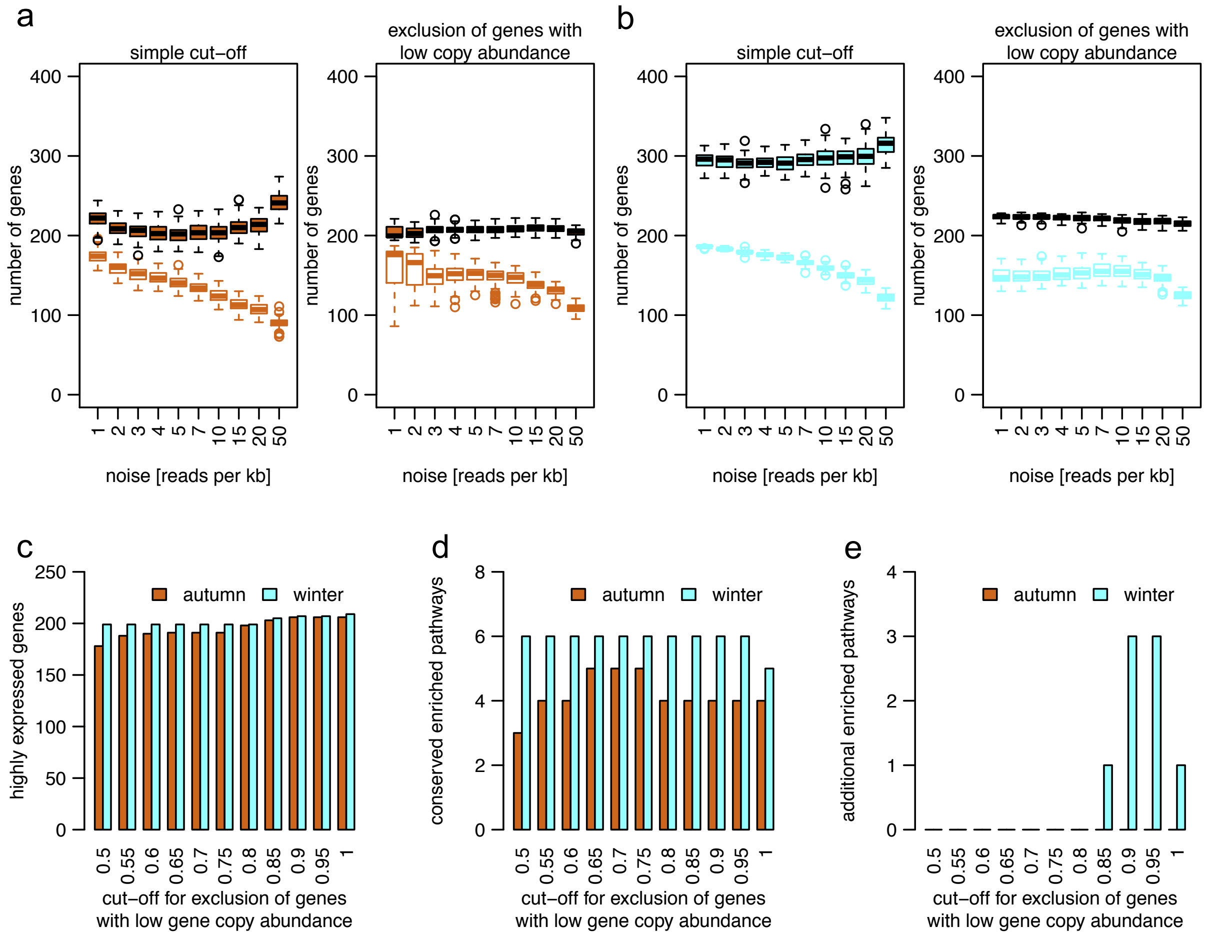

Supplementary Figure 3 Results of the sensitivity analyses. (**a**) and (**b**) Effect of noise on the number and identity of genes with high relative expression determined by comparing a simple numerical cut-off and our method for the reduction of false-positives by excluding genes with very low gene copy abundances. Filled boxes indicate total number of genes with high relative expression, boxes with blue or brown lines indicate the sizes of the intersect of genes with high relative expression without noise and after addition of noise; (**a**) autumn dataset, (**b**) winter dataset. (**c**) Effect of changing the cut-offs for exclusion of genes with low gene copy abundances to reduce false-positives on numbers of genes with a high relative expression. (**d**) and (**e**) Effect of changing the cut-offs for exclusion of genes with low gene copy abundances to reduce false-positives on the identities of pathways enriched with highly expressed genes. (**d**) Number of pathways enriched with highly expressed genes that are found at the chosen cut-off (0.75) and after variation of the cut-off. (**e**) Number of additional

pathways enriched in highly expressed genes, which are not found at the chosen cut-off but after relaxation of the cut-off.

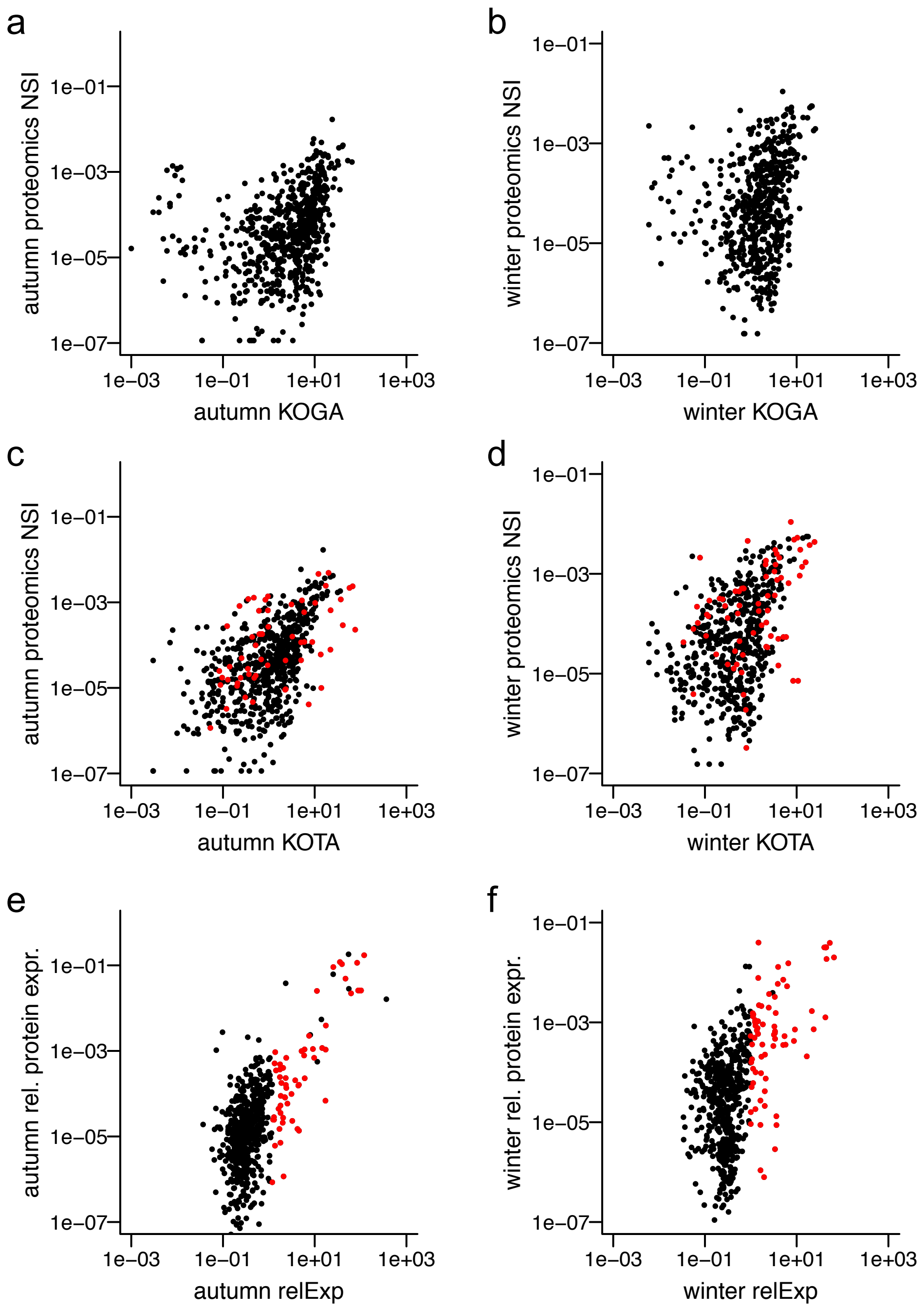

Supplementary Figure 4 Expression of KOs in metabolic pathways at the protein level. (**a**) and (**b**) Comparison of gene copy abundances (*KOGA*) and protein abundances (*NSI*) in the (**a**) autumn and (**b**) winter samples. (**c**) and (**d**) Comparison of gene transcript abundances (*KOTA*) and protein abundances (*NSI*) in the (**c**) autumn and (**d**) winter samples. (**e**) and (**f**) Comparison of expression values relative to gene copy numbers (*KOGA*), transcript expression levels (*KOTA*) and protein abundances (rel. protein exp.) in the (**e**) autumn and (**f**) winter samples. (**c** to **f**) Highly expressed KOs are highlighted in red.

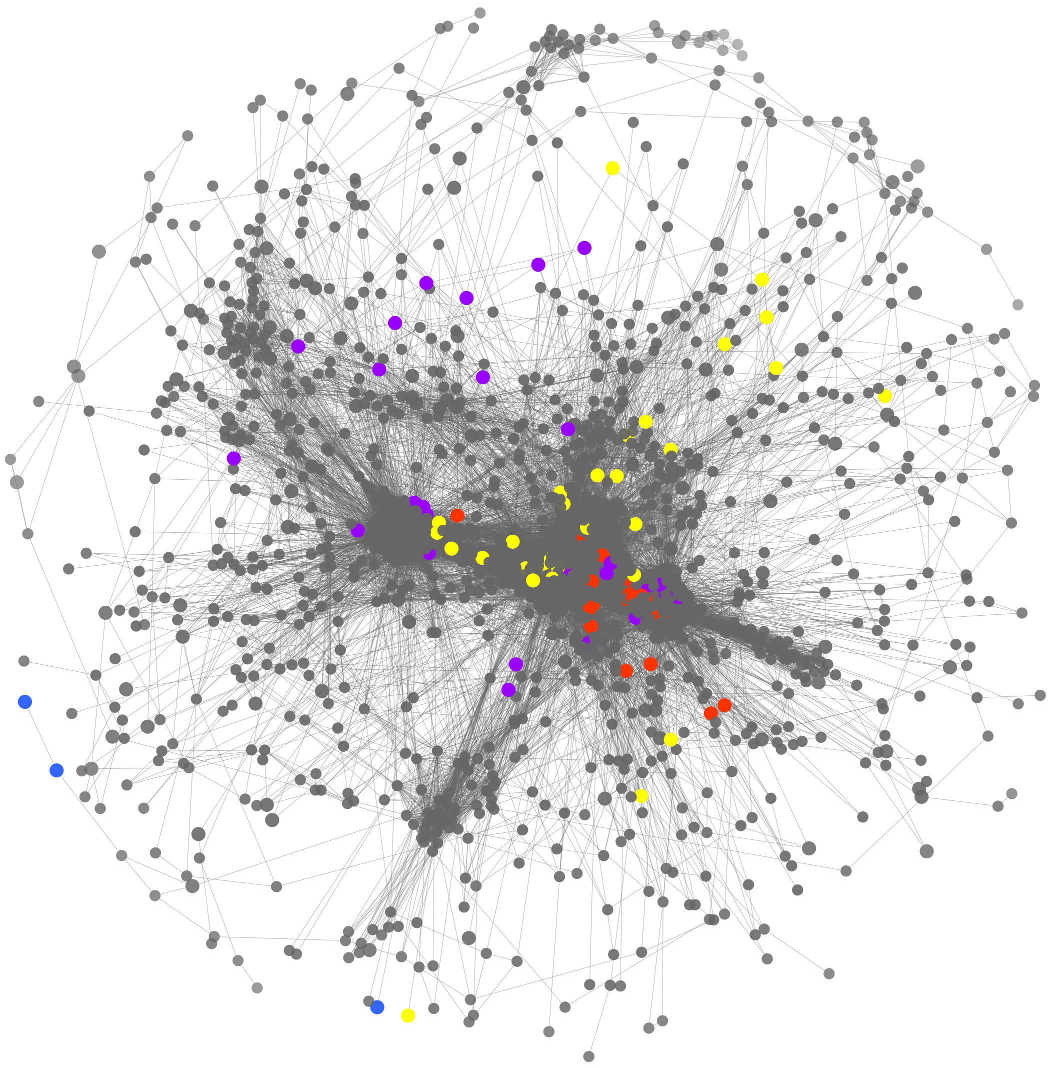

Supplementary Figure 5 Generalized OMMC-wide metabolic network reconstructed from the combined metagenomic and metatranscriptomic data of OMMCs sampled in autumn and winter. Coloured nodes indicate pathways enriched in highly expressed KOs; blue – oxidative phosphorylation; yellow – nitrogen metabolism; red – TCA cycle; purple – glycerolipid metabolism. Large grey nodes are highly expressed during at least one season. Opacity of nodes indicates shortest average path length (the more transparent, the longer the path length).

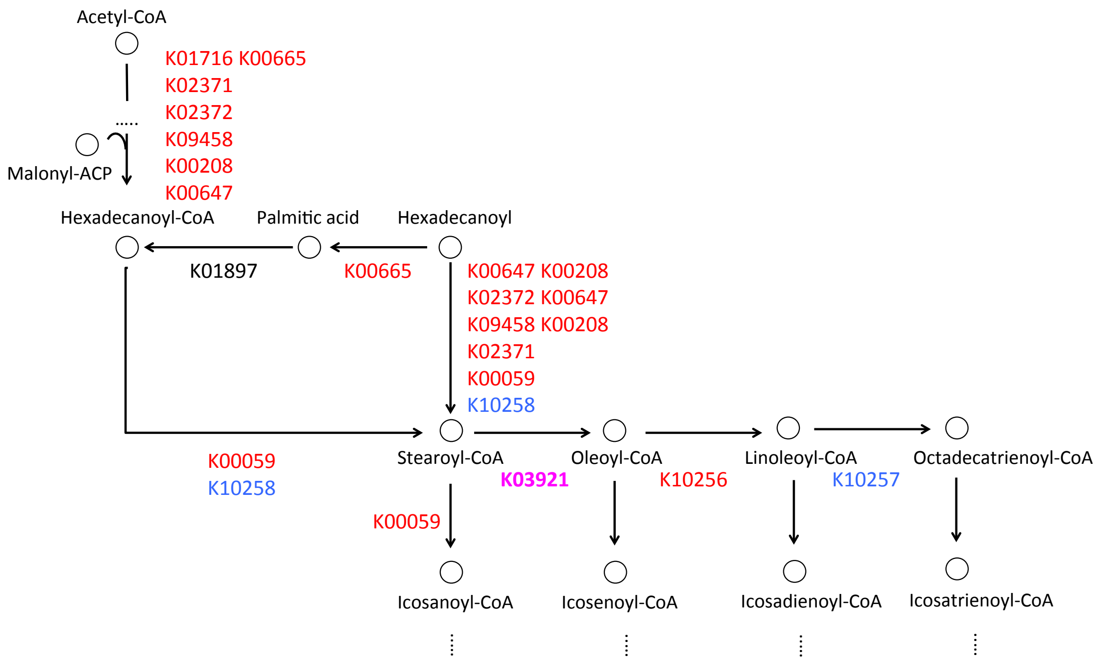

Supplementary Figure 6 Representation of the fatty acid metabolic pathway (ko01212). KOs with a betweenness centrality that is much higher in the metabolic network reconstructed from the OMMC sampled in winter compared to the OMMC sampled in autumn are highlighted in red. The key functionality of KO K03921 (acyl-[acyl-carrier protein] desaturase) is highlighted in pink. Winter KOs with a high relative gene expression are represented in blue. The dotted lines represent the continuity of the pathway and the circles represent metabolites.

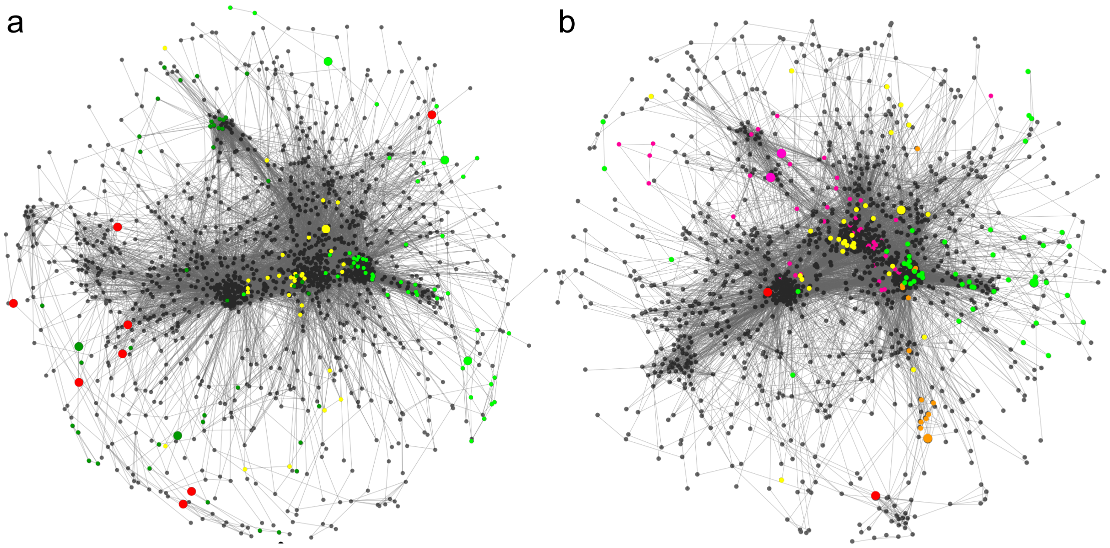

Supplementary Figure 7 OMMC-wide metabolic networks reconstructed from metagenomic and metatranscriptomic data of OMMCs sampled in (**a**) autumn and (**b**) winter. Large nodes indicate KOs encoding key functionalities whereas colours represent pathway membership: yellow, nitrogen metabolism; orange, fatty acid biosynthesis; light green, benzoate degradation; dark green, porphyrin and chlorophyll metabolism; pink, cystein and methionine metabolism and red, other pathways.

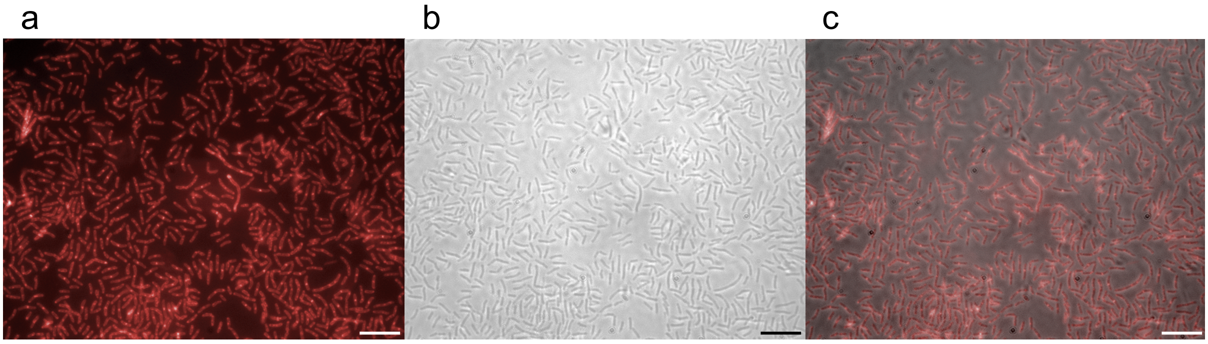

Supplementary Figure 8 Micrographs of Isolate LCSB065. (**a**) Non-polar granules observed in Isolate LCSB065 following Nile Red staining (λ_{ex} 550/20 nm, λ_{em} 620/60 nm); (**b**) Bright field micrograph; (**c**) Overlay of (**a**) and (**b**). The scale bar is equivalent to 10 µm.

Supplementary Tables

Supplementary Table 1 Physicochemical characteristics of the wastewater at the time of sampling in the anoxic tank of the Schifflange BWWT plant.

Sampling dates	Suspended solids (g/l)	pH	NO_3^- (mg/l)	O_2 (mg/l)	NH_4 (mg/l)	PO_4 (mg/l)	Air temperature (°C)	Water temperature (°C)
4 October 2010	2.78	6.94	2.77	0.64	0.6	2.06	15	20.7
25 January 2011	3.24	7.01	3.37	1.13	1.72	1.02	0	14.5

Supplementary Table 2 Quantitative and qualitative analyses of biomacromolecular fractions sequentially isolated from the OMMC samples.

Sampling dates	DNA		RNA		Protein
	A260/A280	Quantity [µg]	RIN	Quantity [µg]	Quantity per gel lane [µg]
4 October 2010	2.20	15.44	8.9	81.97	20.1
25 January 2011	2.03	6.11	9.2	75	21.8

Supplementary Table 3 Statistics of the combined assembly of the metagenomic and metatranscriptomic sequence datasets.

Statistic	Value	Unit
Total contig length	1 617 735 059	nt
Average contig length	239	nt
N50	258	nt
Maximal contig length	12 591	nt
Number of contigs	6 761 781	

Supplementary Table 4 Ammonia-oxidizing organisms with corresponding accession numbers of *amoA* genes used for the reconstruction of the phylogenetic tree.

<table>
<tr><th>Organism name</th><th>Accession number</th></tr>
<tr><td>Candidatus Nitrososphaera gargensis</td><td>gi|166007511</td></tr>
<tr><td>Methylobacter tundripaludum</td><td>gi|493947876</td></tr>
<tr><td>Methylococcus capsulatus Bath</td><td>gi|53803062</td></tr>
<tr><td>Methylomicrobium alcaliphilum 20Z</td><td>gi|357403888</td></tr>
<tr><td>Nitrosococcus halophilus Nc4</td><td>gi|292490804</td></tr>
<tr><td>Nitrosococcus oceani ATCC19707</td><td>gi|77165960</td></tr>
<tr><td>Nitrosococcus watsonii C-113</td><td>gi|300113334</td></tr>
<tr><td>Nitrosomonas cryotolerans ATCC49181</td><td>gi|12620331</td></tr>
<tr><td>Nitrosomonas europaea ATCC19718</td><td>gi|30248947</td></tr>
<tr><td>Nitrosomonas eutropha C91</td><td>gi|114332043</td></tr>
<tr><td>Nitrosomonas sp. Is79A3</td><td>gi|339481967</td></tr>
<tr><td>Nitrosomonas sp. JL21</td><td>gi|19310210</td></tr>
<tr><td>Nitrosomonas sp. AL212</td><td>gi|325981491</td></tr>
<tr><td>Nitrosospira briensis C-128</td><td>gi|1732262</td></tr>
<tr><td>Nitrosospira multiformis ATCC25196</td><td>gi|82701932</td></tr>
<tr><td>Nitrosospira sp. APG3A</td><td>gi|490283770</td></tr>
<tr><td>Nitrosospira sp. En13</td><td>gi|121483569</td></tr>
<tr><td>Nitrosospira sp. NpAV</td><td>gi|2062746</td></tr>
<tr><td>Nitrosospira sp. Np39-19</td><td>gi|2425028</td></tr>
<tr><td>Nitrosovibrio tenuis</td><td>gi|1732264</td></tr>
</table>

Supplementary Table 5 Numbers of metagenomic and metatranscriptomic reads from autumn and winter dates mapping to the isolate genomes of *Nitrosomonas* sp. Is79 (ref. 30) and Isolate LCSB065.

Dataset	Sampling dates	Target organism	
		Nitrosomonas sp. IS79	Isolate LCSB065
metagenomic reads	4 October 2010	7 282	5 665
metagenomic reads	25 January 2011	13 058	7 941
metatranscriptomic reads	4 October 2010	4 004	1 923
metatranscriptomic reads	25 January 2011	28 631	20 784

Supplementary References

1. Roume H, Muller EE, Cordes T, Renaut J, Hiller K, Wilmes P. A biomolecular isolation framework for eco-systems biology. *ISME J* 2013; **7:** 110-121.

2. Roume H, Heintz-Buschart A, Muller EE, Wilmes P. Sequential isolation of metabolites, RNA, DNA, and proteins from the same unique sample. Microbial Metagenomics, Metatranscriptomics, and Metaproteomics. *Method Enzymol* 2013; **531:** 219-236.

3. Kozarewa I, Turner DJ. 96-plex molecular barcoding for the Illumina Genome Analyzer. *High-Throughput Next Generation Sequencing* 2011; 279-298.

4. Masella AP, Bartram AK, Truszkowski JM, Brown DG, Neufeld JD. PANDAseq: paired-end assembler for illumina sequences. *BMC Bioinformatics* 2012; **13:** 31.

5. Kofler R, Orozco-terWengel P, De Maio N, Pandey RV, Nolte V, Futschik A *et al.* PoPoolation: a toolbox for population genetic analysis of next generation sequencing data from pooled individuals. *PloS one* 2011; **6:** e15925.

6. Huang Y, Niu B, Gao Y, Fu L, Li W. CD-HIT Suite: a web server for clustering and comparing biological sequences. *Bioinformatics* 2010; **26:** 680-682.

7. Kultima JR, Sunagawa S, Li J, Chen W, Chen H, Mende DR *et al.* MOCAT: a metagenomics assembly and gene prediction toolkit. *PloS one* 2012; **7:** e47656.

8. Li R, Li Y, Kristiansen K, Wang J. SOAP: short oligonucleotide alignment program.

Bioinformatics 2008; **24:** 713-714.

9. Miller CS, Baker BJ, Thomas BC, Singer SW, Banfield JF. EMIRGE: reconstruction of full-length ribosomal genes from microbial community short read sequencing data. *Genome Biol* 2011; **12:** R44.

10. Quast C, Pruesse E, Yilmaz P, Gerken J, Schweer T, Yarza P *et al.* The SILVA ribosomal RNA gene database project: improved data processing and web-based tools. *Nucleic Acids Res* 2013; **41:** D590-D596.

11. Treangen TJ, Koren S, Sommer DD, Liu B, Astrovskaya I, Ondov B *et al.* MetAMOS: a modular and open source metagenomic assembly and analysis pipeline. *Genome Biol* 2013; **14:** R2.

12. Namiki T, Hachiya T, Tanaka H, Sakakibara Y. MetaVelvet: an extension of Velvet assembler to de novo metagenome assembly from short sequence reads. *Nucleic Acids Res* 2012; **40:** e155-e155.

13. Rho M, Tang H, Ye Y. FragGeneScan: predicting genes in short and error-prone reads. *Nucleic Acids Res* 2010; **38:** e191-e191.

14. Hyatt D, Chen G-L, LoCascio P, Land M, Larimer F, Hauser L. Prodigal: prokaryotic gene recognition and translation initiation site identification. *BMC Bioinformatics* 2010; **11:** 119.

15. Kent WJ. BLAT—the BLAST-like alignment tool. *Genome Res* 2002; **12:** 656-664.

16. Kessner D, Chambers M, Burke R, Agus D, Mallick P. ProteoWizard: open source software for rapid proteomics tools development. *Bioinformatics* 2008; **24:** 2534-2536.

17. Craig R, Cortens JP, Beavis RC. Open source system for analyzing, validating, and storing protein identification data. *J Proteome Res* 2004; **3:** 1234-1242.

18. Deutsch EW, Mendoza L, Shteynberg D, Farrah T, Lam H, Tasman N *et al.* A guided tour of the Trans-Proteomic Pipeline. *Proteomics* 2010; **10:** 1150-1159.

19. Keller A, Nesvizhskii AI, Kolker E, Aebersold R. Empirical statistical model to estimate the accuracy of peptide identifications made by MS/MS and database search. *Anal Chem* 2002; **74:** 5383-5392.

20. Shteynberg D, Deutsch EW, Lam H, Eng JK, Sun Z, Tasman N *et al.* iProphet: multi-level integrative analysis of shotgun proteomic data improves peptide and protein identification rates and error estimates. *Mol Cell Proteomics* 2011; **10:** M111.007690.

21. Muller EE, Pinel N, Laczny CC, Hoopmann MR, Narayanasamy S, Lebrun LA *et al.* Community-integrated omics links dominance of a microbial generalist to fine-tuned resource usage. *Nat Commun* 2014; **5:** 5603; 1-10.

22. Lee S, Seo CH, Lim B, Yang JO, Oh J, Kim M *et al.* Accurate quantification of transcriptome from RNA-Seq data by effective length normalization. *Nucleic Acids Res* 2011;

39: e9-e9.

23. Tsementzi D, Poretsky R, Rodriguez-R LM, Luo C, Konstantinidis KT. Evaluation of metatranscriptomic protocols and application to the study of freshwater microbial communities. *Environ Microbiol Rep* 2014; **6:** 640-655.

24. Benjamini Y, Hochberg Y. Controlling the false discovery rate: a practical and powerful approach to multiple testing. *Journal of the Royal Statistical Society. Series B* (*Methodological)* 1995; 289-300.

25. Shannon P, Markiel A, Ozier O, Baliga NS, Wang JT, Ramage D *et al.* Cytoscape: a software environment for integrated models of biomolecular interaction networks. *Genome Res* 2003; **13:** 2498-2504.

26. Rahman SA, Schomburg D. Observing local and global properties of metabolic pathways:'load points' and 'choke points' in the metabolic networks. *Bioinformatics* 2006; **22:** 1767-1774.

27. Faust K, Croes D, van Helden J. Metabolic pathfinding using RPAIR annotation. *J Mol Biol* 2009; **388:** 390-414.

28. Csardi G, Nepusz T. The igraph software package for complex network research. *InterJournal, Complex Systems* 2006; **1695:** 1-9.

29. Altschul SF, Gish W, Miller W, Myers EW, Lipman DJ. Basic local alignment search

tool. *J Mol Biol* 1990; **215:** 403-410.

30. Bollmann A, Sedlacek CJ, Norton J, Laanbroek HJ, Suwa Y, Stein LY *et al.* Complete genome sequence of *Nitrosomonas* sp. Is79, an ammonia oxidizing bacterium adapted to low ammonium concentrations. *Stand Genomic Sci* 2013; **7:** 469.

31. Liu W, Li L, Khan MA, Zhu F. Popular molecular markers in bacteria. *Mol Genet Microbiol Virol* 2012; **27:** 103-107.

32. Sommer DD, Delcher AL, Salzberg SL, Pop M. Minimus: a fast, lightweight genome assembler. *BMC Bioinformatics* 2007; **8:** 64.

33. Treangen TJ, Sommer DD, Angly FE, Koren S, Pop M. Next generation sequence assembly with AMOS. *Curr Protoc Bioinformatics* 2011; 11.8. 1-11.8. 18.

34. Sayavedra-Soto L, Hommes N, Alzerreca J, Arp D, Norton JM, Klotz M. Transcription of the amoC, amoA and amoB genes in *Nitrosomonas europaea* and *Nitrosospira* sp. NpAV. *FEMS Microbiol Lett* 1998; **167:** 81-88.

35. Fu L, Niu B, Zhu Z, Wu S, Li W. CD-HIT: accelerated for clustering the next-generation sequencing data. *Bioinformatics* 2012; **28:** 3150-3152.

36. Dereeper A, Guignon V, Blanc G, Audic S, Buffet S, Chevenet F *et al.* Phylogeny. fr: robust phylogenetic analysis for the non-specialist. *Nucleic Acids Res* 2008; **36:** W465-W469.

37. Castresana J. Selection of conserved blocks from multiple alignments for their use in phylogenetic analysis. *Mol Biol Evol* 2000; **17:** 540-552.

38. Guindon S, Dufayard J-F, Lefort V, Anisimova M, Hordijk W, Gascuel O. New algorithms and methods to estimate maximum-likelihood phylogenies: assessing the performance of PhyML 3.0. *Syst Biol* 2010; **59:** 307-321.

39. Chevenet F, Brun C, Bañuls A-L, Jacq B, Christen R. TreeDyn: towards dynamic graphics and annotations for analyses of trees. *BMC Bioinformatics* 2006; **7:** 439.

40. Reasoner D, Geldreich E. A new medium for the enumeration and subculture of bacteria from potable water. *App Environ Microbiol* 1985; **49:** 1-7.

41. Levantesi C, Rossetti S, Thelen K, Kragelund C, Krooneman J, Eikelboom D *et al.* Phylogeny, physiology and distribution of '*Candidatus* Microthrix calida', a new Microthrix species isolated from industrial activated sludge wastewater treatment plants. *Environ Microbiol* 2006; **8:** 1552-1563.

42. Slijkhuis H. Microthrix parvicella, a filamentous bacterium isolated from activated sludge: cultivation in a chemically defined medium. *App Environ Microbiol* 1983; **46:** 832-839.

43. Fowler SD, Greenspan P. Application of Nile red, a fluorescent hydrophobic probe, for the detection of neutral lipid deposits in tissue sections: comparison with oil red O. *J Histochem Cytochem* 1985; **33:** 833-836.

44. Schneider CA, Rasband WS, Eliceiri KW. NIH Image to ImageJ: 25 years of image analysis. *Nat Methods* 2012; **9:** 671-675.

45. Rodrigue S, Materna AC, Timberlake SC, Blackburn MC, Malmstrom RR, Alm EJ, Chisholm SW. Unlocking short read sequencing for metagenomics. *PLoS one* 2010; **5:** e11840.

46. Xu H, Luo X, Qian J, Pang X, Song J, Qian G *et al.* FastUniq: a fast de novo duplicates removal tool for paired short reads. *PloS one* 2012; **7:** e52249.

47. Peng Y, Leung HC, Yiu S-M, Chin FY. IDBA-UD: a de novo assembler for single-cell and metagenomic sequencing data with highly uneven depth. *Bioinformatics* 2012; **28:** 1420-1428.

48. Gordon D, Abajian C, Green P. Consed: a graphical tool for sequence finishing. *Genome Res* 1998; **8:** 195-202.

49. Aziz RK, Bartels D, Best AA, DeJongh M, Disz T, Edwards RA *et al.* The RAST Server: rapid annotations using subsystems technology. *BMC Genomics* 2008; **9:** 75.

50. Li H, Durbin R. Fast and accurate short read alignment with Burrows–Wheeler transform. *Bioinformatics* 2009; **25:** 1754-1760.

51. Kerepesi C, Bánky D, Grolmusz V. AmphoraNet: The webserver implementation of the AMPHORA2 metagenomic workflow suite. *Gene* 2014; **533:** 538-540.

52. Tatusov RL, Galperin MY, Natale DA, Koonin EV. The COG database: a tool for genome-scale analysis of protein functions and evolution. *Nucleic Acids Res* 2000; **28:** 33-36.

53. Overbeek R, Begley T, Butler RM, Choudhuri JV, Chuang H-Y, Cohoon M *et al.* The subsystems approach to genome annotation and its use in the project to annotate 1000 genomes. *Nucleic Acids Res* 2005; **33:** 5691-5702.
